# Expression of the Arabidopsis redox-related LEA protein, *SAG21* is regulated by ERF, NAC and WRKY transcription factors

**DOI:** 10.1038/s41598-024-58161-0

**Published:** 2024-04-02

**Authors:** Kelly V. Evans, Elspeth Ransom, Swapna Nayakoti, Ben Wilding, Faezah Mohd Salleh, Irena Gržina, Lieselotte Erber, Carmen Tse, Claire Hill, Krzysztof Polanski, Alistair Holland, Sherien Bukhat, Robert J. Herbert, Barend H. J. de Graaf, Katherine Denby, Vicky Buchanan-Wollaston, Hilary J. Rogers

**Affiliations:** 1 School of Biosciences, Cardiff University, Sir Martin Evans Building, Museum Avenue, Cardiff, CF10 3AT UK; 2 Investigative and Forensic Sciences Research Group, Universiti Teknologi Malaysia, 81310 Johor Bahru, Johor Malaysia; 3 School of Life Sciences, University of Warwick, Coventry, CV4 7AL UK; 4 School of Science and the Environment, University of Worcester, Henwick Grove, Worcester, WR2 6AJ UK; 5 Department of Biology, Centre for Novel Agricultural Products (CNAP), University of York, Heslington, York, YO10 5DD UK

**Keywords:** Abiotic stress, Biotic stress, Ethylene, NAC transcription factors, SAG21/LEA5, WRKY transcription factors, Molecular biology, Plant sciences

## Abstract

SAG21/LEA5 is an unusual late embryogenesis abundant protein in *Arabidopsis thaliana*, that is primarily mitochondrially located and may be important in regulating translation in both chloroplasts and mitochondria. *SAG21* expression is regulated by a plethora of abiotic and biotic stresses and plant growth regulators indicating a complex regulatory network. To identify key transcription factors regulating *SAG21* expression, yeast-1-hybrid screens were used to identify transcription factors that bind the 1685 bp upstream of the *SAG21* translational start site. Thirty-three transcription factors from nine different families bound to the *SAG21* promoter, including members of the ERF, WRKY and NAC families. Key binding sites for both NAC and WRKY transcription factors were tested through site directed mutagenesis indicating the presence of cryptic binding sites for both these transcription factor families. Co-expression in protoplasts confirmed the activation of *SAG21* by WRKY63/ABO3, and *SAG21* upregulation elicited by oligogalacturonide elicitors was partially dependent on WRKY63, indicating its role in *SAG21* pathogen responses. *SAG21* upregulation by ethylene was abolished in the *erf1* mutant, while wound-induced *SAG21* expression was abolished in *anac71* mutants, indicating *SAG21* expression can be regulated by several distinct transcription factors depending on the stress condition.

## Introduction

In their natural environment, plants are subjected to a constantly fluctuating ensemble of abiotic and biotic stressors including rapid or prolonged changes in temperature, water availability, mechanical stress, e.g., wounding, and salinity, as well as attack from herbivores and pathogens. Stress-responsive signalling pathways enable plants to perceive these adverse environmental cues and transmit this information through a network of specific and common signals to help cells withstand short term or longer-term stresses^1^. This signalling induces changes at the molecular (transcriptomic and proteomic), cellular and physiological levels which can be persistent or short lived^2^. Signalling cascades are functionally related, but stressor specific, with responses to multiple stresses differing from responses to individual stresses^3^. However, many different abiotic stresses lead to imbalances in metabolic pathways which induce oxidative stress^4^, caused by the production and accumulation of reactive oxygen species (ROS). This can lead to changes in cellular redox status, direct oxidative modification of components within signalling pathways and oxidative damage to membrane proteins.

Transcriptional activation and repression occur through the binding of transcription factors (TFs) to sequence specific cis-elements within the target gene promoter. A total of 2296 TF genes have been identified within the Arabidopsis genome and classified into 58 families based on their DNA binding domains (Plant RegMap^5^). Of these 58 families, several are particularly involved in stress responses. These include the 71 WRKYs^6,7^, 117 NACs^8^, 122 ERFs^9^, and the 126 MYB family TFs^10^. The cis-elements to which these TFs bind are mostly well-defined, for example W-boxes in the case of WRKY TFs^11^, which share the core sequence TGAC, and the NAC recognition sequence (NACRS) for NAC TFs, although atypical cis-elements are also found^12^. Typically, ERF/AP2 family TFs bind to DRE/CRT promoter elements to activate abiotic stress responses and GCC-boxes to regulate biotic stress resistance^9^ although again divergent ERF TF binding motifs are also found.

Many WRKY TFs are part of biotic and abiotic stress signalling networks. At least eight Arabidopsis WRKY TFs are upregulated by reactive oxygen species^6^, and at least another six Arabidopsis WRKY TFs are activated by drought stress^13^. Interestingly, two WRKY TF genes, *AtWRKY40* and *AtWRKY63* regulate stress-responsive mitochondrial genes^14^ but not mitochondrial genes that are constitutively expressed. WRKY TFs are divided into three phylogenetically related groups (I–III) and group III is further divided into IIIa and IIIb^15^. All the WRKY TFs contain a highly conserved 60 amino acid sequence, the WRKY domain, required for binding target promoters. NAC TF genes also respond to both abiotic and biotic stresses and some are also responsive to ROS^16^.

Downstream of the TF networks, a plethora of genes activate responses to environmental and developmental signals, although relatively few TF targets have been verified experimentally. Late embryogenesis abundant (LEA) proteins form a family of 51 members divided into nine groups, many of which accumulate in response to abiotic stresses including osmotic stress, cold, drought, salinity and freezing^17^. Many have been demonstrated to be functionally important in stress responses as their over- and/or ectopic expression results in stress protection^18^. They are small (10–30 kD) hydrophilic proteins and were first discovered as accumulating in and during seed desiccation, although they are also expressed in vegetative and floral organs^19^. Although their precise role in plant cells remains largely undiscovered, evidence suggests that they are involved in regulating stress tolerance to cellular dehydration, through the stabilisation of cell membranes, prevention of protein aggregation, nucleic acid homeostasis and redox balancing^20–22^.

Senescence Associated Gene 21 (*SAG21/AtLEA5//LEA38*; At4g02380) is a member of the Group 3 LEA proteins^23^. It is mitochondrially located^24^ in root cells, although it has also been detected in chloroplasts of Arabidopsis leaf protoplasts^25^, unlike its paralogue (LEA2; At1g02820) which is located in the cytosol. Three gene models have been predicted for the SAG21 protein (TAIR; Supplementary Fig. 1A) and have been identified as transcripts (TAIR^26^). Unfortunately, proteomic analysis (http://www.peptideatlas.org/) does not extend far enough towards the N terminal to be able to discriminate between the three models, therefore it is not clear whether all three are transcribed in all tissues where the gene is expressed and or translated. SAG21/LEA5 was identified in a complementation screen of the Δ yap1 oxidant sensitive yeast mutant^27^, suggesting that a key role for SAG21 may be in protection against oxidative stress and reactive oxygen species. Very recently a function for SAG21 in regulating translation in both mitochondria and chloroplasts has been reported which may be a key mechanism for its role in protection against stress^25^.

The *SAG21* gene was originally identified as being up-regulated during leaf senescence, peaking in abundance prior to full senescence then declining with or shortly after the onset of visible senescence^28^. However, it is also highly expressed in pollen, petals, and roots^24,27^. As well as developmental regulation, *SAG21* expression is up-regulated in response to a variety of abiotic and biotic stresses including cold^29^, dehydration^30^, salinity, and wounding^24^, phloem feeders^31^, *Botrytis cinerea* infection^24^ and both fungal and bacterial elicitors^32^ as well as being light regulated^24,27^. *SAG21* was also identified in a genome wide association study as associated with adaptation to environmental stress^33^. Moreover, *SAG21* expression is up-regulated by several stress-related plant growth regulators including ethylene, abscisic acid (ABA), jasmonic acid (JA) and salicylic acid (SA) as well as ROS^24,27^ which may be mediating all or part of the response to stresses. However, although induction of *SAG21* in response to dehydration is dependent upon ABA synthesis it is independent of the protein phosphatase ABI1^27^, a key mediator in ABA response pathways. Many LEA proteins can be induced by combinations of several different stresses and stress-related hormones. For example, *ABR* (*ABA-response protein*) which belongs to the LEA_4 family is induced by ABA, NaCl, mannitol and darkness^34^ and the bZIP transcription factor ABI5 binds directly to G-boxes in its promoter.

Given the complexity of the *SAG21* responses to the environment and through development, an analysis of its promoter was undertaken using a yeast-1-hybrid (Y1H) screening approach to identify potential TF regulators. Their functional role was then validated by their co-expression in protoplasts with a SAG21p-GUS reporter and analysis of *SAG21* expression in mutants. Results show that a wide range of TFs can bind to the *SAG21* promoter, and identifies specific members of the *ERF*, *WRKY* and *NAC* TF gene families that are functionally important for *SAG21* response to environmental signals.

## Results

### Gene model 1 for *SAG21* seems prevalent in Arabidopsis seedlings

For analysis of the *SAG21* promoter region it was first important to define the correct translational start point. The three gene models for AT4G02380 (*SAG21/LEA5*) predict proteins of three different lengths, one of which (model 2) includes an alternative splice site (Supplementary Fig. 1A). The longest, model 3, includes 359 bp of coding sequence between the intron and the ATG start codon while models 1 and 2 include 74 and 13 bp respectively (Supplementary Fig. 1A) resulting in predicted proteins of 192, 97 and 78 amino acids respectively. To test whether mRNAs consistent with the longest two models were expressed in seedlings, primers were designed to span the ATG start codon in models 1 and 3 and a downstream sequence within the open reading frame. They were tested both on cDNA and genomic DNA. Amplification of a 514 bp PCR product with genomic DNA as template but absence of the expected 414 bp product from cDNA template confirms that, at least in seedlings, both grown under optimal conditions, and stressed, mRNA consistent with model 3 is absent (Supplementary Fig. 1B,C). However, primers spanning the start codon of model 1 and including the intron produced the expected 216 bp product with genomic DNA and the smaller 116 bp product with the cDNA confirming that an mRNA consistent with model 1 is present. An alignment of the model 3 predicted protein with orthologous genes confirms that in other species homology extends up to start of the model 1 predicted sequence, supporting model 1 as likely translated product. Model 1 was therefore used for analysis of the promoter region.

### Co-expression of *SAG21* with transcription factors, binding of TFs to the *SAG21* promoter revealed by ChIP and predicted distribution of cis-elements on the *SAG21* promoter

A first analysis of the TFs that might regulate *SAG21* was performed using co-expression analysis in PlantPAN3.0. This revealed a high Pearson correlation coefficient (> 0.8) for five TFs linked to development, each from a different family, and 35 TFs from 17 different families linked to stress responses (Supplementary Table 1). Most highly represented TF families associated with stress were Dof (5 TFs) followed by NAC (4), WRKY, MADS and GATA (3). ChIP experiments were also analysed in PlantPAN3.0 to identify TFs known to bind to the 1685 bp upstream of the *SAG21* translational start site based on model 1 above. This analysis identified 52 TFs across 32 ChIP experiments. The highest number (21) were identified in an ABA response experiment, 18 TFs in 17 different experiments with seedlings, and eight related to flowering (Supplementary Table 2).

**Figure 1:**
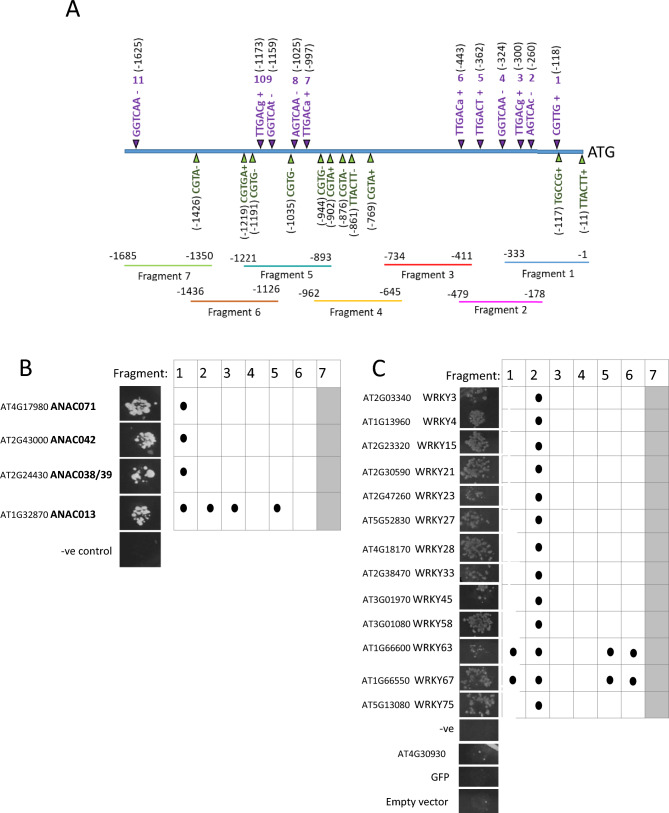
Analysis of the *SAG21* promoter for NAC and WRKY TF binding. (**A**) Position and sequence of W-boxes (numbered 1–11, position in brackets, in purple) and ANAC TF binding sites (below in green) on the 1685 bp upstream of the translational start of *SAG21* based on the PlantPAN 2.0 database, Lindemose et al.^35^ and Wu et al.^36^; uppercase letters indicate canonical sequence. Below, the seven fragments used for a yeast-1-hybrid screen of the WRKY transcription factor library. Positively identified NAC (**B**) and WRKY (**C**) transcription factors interacting with the *SAG21* promoter fragments based on yeast-1-hybrid interactions observed as growth on SD-LTH medium. Promoter fragments resulting in autoactivation of the reporter gene are shaded in grey.

To gain a better overview of TFs that might bind to specific regions of the *SAG21* promoter, the 1685 bp upstream of the *SAG21* translational start site (based on model 1) were divided into seven overlapping fragments (Fig. 1A). Using PlantPAN3.0, each of the seven overlapping fragments were assessed for the presence of known cis-elements that potentially bind TF families (Table 1), considering the unique portions of each fragment and the overlapping portions separately. Fragment 7 and Fragment 3 contained cis-elements for the largest number of TF families (27 and 22 respectively) while Dof, GATA and ZF-HD families were present on most different portions of the promoter. Cis-elements for 15 different TF families were only found on a single promoter portion.

**Table 1:** TF families predicted to bind to sections of the 1685 bp upstream of *SAG21* translational start site (PlantPAN3.0).

Fragment	F1	F1–F2	F2	F2–F3	F3	F3–F4	F4	F4–F5	F5	F5–F6	F6	F6–F7	F7
AP2/ERF	+		+				+	+	+		+	+	+
ARID					+	+							+
ARR-B	+		+		+	+	+				+		+
AT-Hook	+	+	+	+	+	+	+		+		+	+	+
B3													+
bHLH									+	+			+
bZIP	+	+			+		+	+	+	+			+
C2H2	+	+	+	+	+		+						+
C3H													+
C3H Zinc finger					+								+
CPP							+						
CSD	+												
Dof	+	+	+	+	+	+	+	+	+	+	+		+
EIL							+						
EIN3							+						
GATA	+	+	+	+	+	+	+	+	+	+	+	+	+
GRF					+								
HB-PHD													+
HD-ZIP					+		+	+	+	+			+
Homeodomain		+	+	+			+	+					+
MADS box													+
M-type													+
MYB			+		+	+	+						+
Myb/SANT	+		+		+	+	+						+
MYB-related					+				+				
NAC		+	+					+					+
NAM		+	+				+	+					
NF-YA			+		+				+	+	+	+	+
NF-YB			+		+				+	+	+	+	+
NF-YC			+		+				+	+	+	+	+
RAV													+
SBP									+				
Sox	+		+		+	+							
TALE		+		+					+	+			+
TBP		+	+		+	+	+				+		
TCR							+						
tify	+	+			+	+	+	+	+	+		+	
Trihelix											+		
WOX							+	+					
WRC					+								
WRKY		+	+						+	+			+
YABBY	+		+		+	+	+				+	+	
ZF-HD	+	+	+	+	+	+	+	+	+		+	+	+
Total	13	13	19	7	22	12	21	11	16	11	12	9	27

Similar TF families were identified from the three types of analyses but there was remarkably little overlap in the specific TFs identified through co-expression analyses and from ChiP datasets. An experimental approach was therefore employed to complement the bioinformatic analysis.

### A yeast-1 hybrid screen of 1500 plant transcription factors with *SAG21* gene promoter sequences identified 16 transcription factors

A screen of over 1500 pooled plant TFs using the seven overlapping fragments of the *SAG21* promoter (Fig. 1A), spanning 1685 bp upstream of the gene model 1 ATG resulted in the identification of 58 different TFs from 22 families. The ERF family represented the largest group (11 members were identified) followed by HDZIP and TCP (9 members of each) (Supplementary Table 3). Fragment 5 of the *SAG21* promoter (− 893 to − 1221) bound most TFs (32) followed by Fragment 1 (-1 to -333) which bound 14, while no TFs bound to Fragment 7, although for this fragment there was substantial autoactivation by the promoter fragment in the absence of the TFs which may have obscured genuine binding.

To confirm the TF-SAG21 promoter interactions following the library screen, 21 individual TFs were paired with the *SAG21* promoter fragments. TFs were selected on the basis of their identification in the library screen and availability of the TF in the Y1H vector. Of these, 16 were confirmed to bind to the proximal six *SAG21* promoter fragments (Fragments 1 to 6; Table 2, Supplementary Fig. 2). Many of the TFs bound to more or different fragments than the one that they bound to in the initial screen (Supplementary Table 3). Eight of the TFs tested only bound to one fragment, two bound to two fragments and four TFs bound to three promoter fragments. Of the 16 TFs binding to the *SAG21* promoter, five were from the ERF family, five were homeodomain leucine zipper TFs, two were TCP TFs and the remainder were one each of bZIP, zinc finger, ARID and MYB families. All the TF families identified and confirmed as Y1H interactors were represented in the predicted cis-elements except the TCP family.

**Table 2:** TFs binding to the seven fragments of the *SAG21* 1685 bp promoter region identified by the Y1H screen of pools of 1500 TFs.

AT code	TF name	Fragment						
		1	2	3	4	5	6	7*
AT3G23240	ERF1	●	●			●		
AT5G47230	ERF5				●			
AT1G03800	ERF10					●		
AT1G04370	ERF14			●	●	●		
AT2G31230	ERF15	●						
AT3G15030	TCP4			●		●	●	
AT1G58100	TCP8			●		●	●	
AT5G65310	HB5			●				
AT3G61890	HB12			●		●		
AT1G69780	HB13			●		●		
AT5G39760	HB23			●	●	●		
AT5G53980	HB52			●				
AT3G19290	ABF4					●		
AT1G27730	ZAT10/STZ		●					
AT1G76110	HMG-Box		●	●				
AT3G12720	MYB67					●		

*Autoactivation of fragment 7 made it impossible to detect binding.

### Four NAC TFs bind to the SAG21 promoter by Y1H

The *SAG21* promoter contains at least 11 NAC TF predicted binding sites (Fig. 1A) based on the canonical CGT[G*/*A] sequence and other *SAG21* promoter sequences were identified as potential binding sequences of other NAC TFs: including TT(A/C/G)CTT^35^ and TGCC[GT]^36^, although more cryptic sites may be present. Two NAC TFs (*ANAC102* and *ANAC038*), were identified in the initial screen of 1500 TFs (which included 62 NAC TFs). Both bound to Fragment 5 (Supplementary Table 3), but neither were confirmed as binding to this fragment of the *SAG21* promoter when re-tested. A separate Y1H screen of 94 Arabidopsis NAC TFs only was therefore performed and identified three NAC TFs that bound only to Fragment 1 of the *SAG21* promoter: *ANAC042*, *ANAC071* and *ANAC038/39,* while *ANAC013* bound to four different fragments, including Fragment 1 (Fig. 1B).

**Figure 2:**
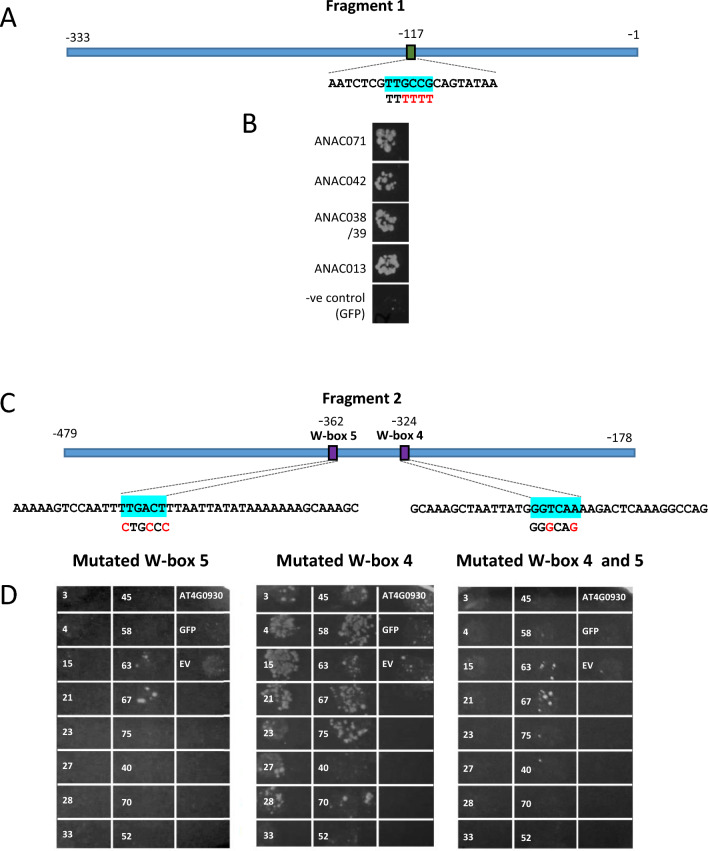
Effect of mutating *SAG21* promoter cis elements identified as biding sites for (**A,B**) NAC and (**C,D**) WRKY TFs. Regions highlighted in blue represent the W-box sequence, with bases in red below indicating the mutated bases. (**B,D**) Interactions observed on SD-LTH medium from a Y1H assay of (**B**) NAC TFs binding to fragment 1 and (**D**) WRKY TFs binding to fragment 2 and negative controls, using fragment 2 containing mutated W-box 5, mutated W-box 4 and mutated W-box 4 and 5. WRKY TF number is indicated for each interaction image.

To investigate further the binding of NAC TFs to Fragment 1, the promoter sequence was analysed for the canonical NAC TF recognition sequence CGT[G/A]. This was not present, but a partial match to the RRYGCCGT sequence which is the core ANAC042 binding sequence^36^ was located at − 117 bp upstream from the ATG in the *SAG21* promoter. This GCCGT sequence was mutated (Fig. 2A) and binding of *ANAC042*, *ANAC071, ANAC038/39,* and *ANAC013* was re-tested (Fig. 2B). However, the mutation did not appear to affect binding, indicating that the binding site for these NAC TFs is likely to be different and as yet unidentified.

### Thirteen WRKY transcription factors bind to the *SAG21* promoter

A bioinformatic analysis of the 1685 bp upstream of the functional *SAG21* ATG start codon revealed eleven W boxes, four of which conformed to the canonical (T)TGAC(C/T) sequence (Fig. 1A). Apart from a non-canonical CGTTG at position − 120 from the ATG which is predicted to bind just one WRKY transcription factor (WRKY1) all the others are predicted to bind between 60 and 72 WRKY transcription factors. As no WRKY TFs were found in the 1500 TF screen (although 61 WRKY TFs were included), a second Y1H library containing 68 WRKY TFs was screened separately, using the same seven *SAG21* promoter fragments (Fig. 1A). Based on this Y1H assay, 13 WRKY transcription factors bound to the *SAG21* promoter fragments. This is not totally unexpected as the yeast clones were pooled in the 1500 library and therefore not all may have been isolated from the screen. All 13 WRKY TFs bound to Fragment 2 (− 179 to − 479; Fig. 1C), while WRKY63 and WRKY67 also bound to Fragment 1 (− 1 to − 133), Fragment 5 (− 886 to − 1205) and Fragment 6 (− 1126 to − 1445). No WRKY TF were found in this library to bind to *SAG21* promoter Fragments 3, 4 or 7.

To further identify whether the canonical W-boxes identified in Fragment 2 (Fig. 2C) are binding sites for the WRKY TFs, mutations were introduced into the sequences of W-box 4 (at − 324 from the ATG) and W-box 5 (at − 362 from the ATG) the *SAG21* promoter (Fig. 2C). Mutation of W-box 5 resulted in loss of binding to all of the 13 WRKY TFs except WRKY63 and WRKY67. In contrast, all the 13 previously identified WRKY TFs binding to Fragment 2 interacted with the promoter fragment mutated at W-box 4, except WRKY33 where binding was abolished. WRKY 40, WRKY70 and WRKY52 were included as negative controls as they bound very weakly to the wild type (WT) Fragment 2. Some binding of these TFs is seen to mutated W-box 4 but much weaker than the other WRKY TFs. These results suggest that WRKY33 does not require W-box 4 to bind to the *SAG21* promoter while WRKY63 and WRKY67 do not require W-box 5. The double knockout of W-box 4 and W-box 5 abolished all WRKY-TF interactions, except WRKY63 and WRKY67 (Fig. 2D) suggesting that WRKY63 and WRKY67 may bind to an as yet unidentified cryptic site on Fragment 2 of the *SAG21* promoter.

### Co-expression of SAG21::GUS and pJIT60::WRKY63 in protoplasts increases *SAG21* driven expression

**Figure 3:**
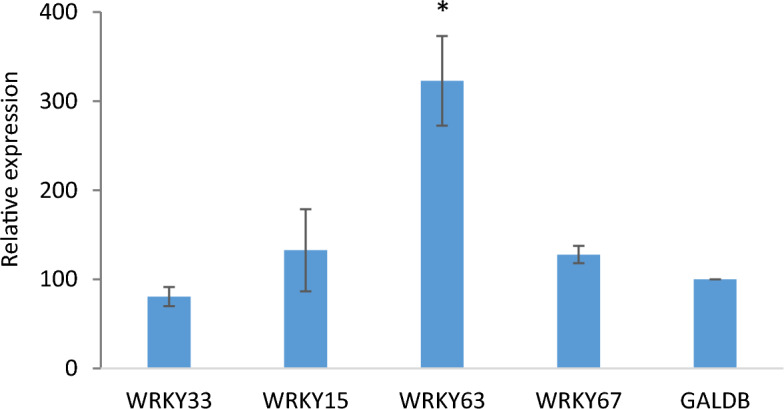
Functional analysis of WRKY-transcription factor interactions. Relative expression levels derived from fluorescence of *SAG21* promoter GUS constructs in response to interactions with transcription factor protein expressing constructs. Both plasmids expressed in Arabidopsis mesophyll protoplasts. The pGPTV-KAN::*SAG21*(1685)::GUS reporter was assayed with pJIT60::WRKY15, pJIT60::WRKY63 and pJIT60::WRKY67 effectors. Relative expression is normalised to LUC and compared to a GALDB control, with the expression value for GALDB arbitrarily set to 100. n = 3, + SE, *indicates significant difference to GALDB (*P* < 0.05) based on a student’s T-test.

To test whether the binding of WRKY TFs to the *SAG21* promoter might be functional in plant cells, Arabidopsis leaf mesophyll protoplasts were co-transformed with a construct in which 1685 bp of the *SAG21* promoter was used to drive expression of GUS, and constructs in which the open reading frames of four WRKY TFs were expressed from the 35S promoter. Four WRKY TFs were selected based on their binding to the *SAG21* promoter fragments in the Y1H screen and their published stress-responsiveness. *WRKY15* expression is up-regulated by reactive oxygen species^37^, WRKY33 appeared to bind specifically to W box 4 on Fragment 2 of the *SAG21* promoter and is involved in abiotic and biotic stress responses^38,39^, WRKY63 and WRKY67 bound to several fragments of the *SAG21* promoter and are involved in responses to drought^40^ and salt^41^ respectively. Co-expression of the *WRKY63* open reading frame with the *SAG21* promoter GUS reporter construct resulted in a significant (*P* < 0.05) more than twofold increase in expression in the protoplasts compared to the GALDB control (Fig. 3). In contrast, co-expression of SAG21p:GUS with *WRKY33*, *WRKY15* or *WRKY67* had no significant effect on *SAG21* driven GUS expression.

### Bioinformatic prediction of upstream regulators of *SAG21*, under stress conditions

**Figure 4:**
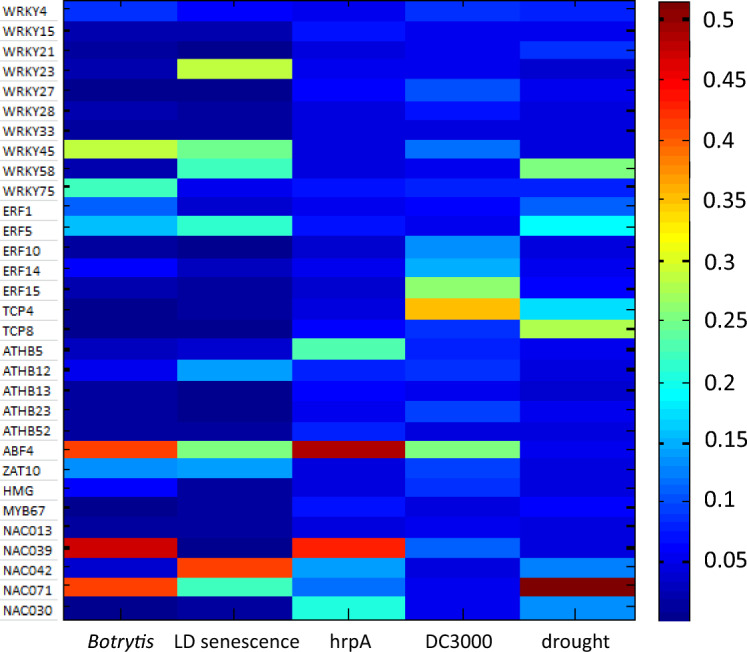
hCSI modelling of *SAG21* and its upstream potential regulators. Heat map shows the probability (maximum possible value = 1) of each TF regulating *SAG21* in a particular stress condition: inoculation with *Botrytis cinerea*, developmental long day (LD) senescence, inoculation with avirulent *hrpA* or virulent DC3000 strains of *Pseudomonas syringae* and drought. Details of treatments in “Methods” section.

hCSI (hierarchical Causal Structure Inference) modelling was used to test whether the TFs identified in the three Y1H screens could be part of potential regulatory networks upstream of *SAG21* when Arabidopsis plants are under four different stress conditions, based on published microarray datasets of their gene expression. Stresses were: inoculation with *Botrytis cinerea*, developmental senescence, inoculation with avirulent *hrpA* or virulent DC3000 strains of *Pseudomonas syringae* and drought. This modelling resulted in the identification of ABF4, and ANAC039 as potential regulators of *SAG21* (probability > 0.4) during both *Botrytis* and *Pseudomonas syringae* infection. ANAC042 was identified as a potential regulator during senescence, whereas ANAC071 may regulate *SAG21* expression both under drought stress and *Botrytis* infection (Fig. 4).

### *SAG21* expression is altered in mutants of ERF, NAC and WRKY transcription factors

**Figure 5:**
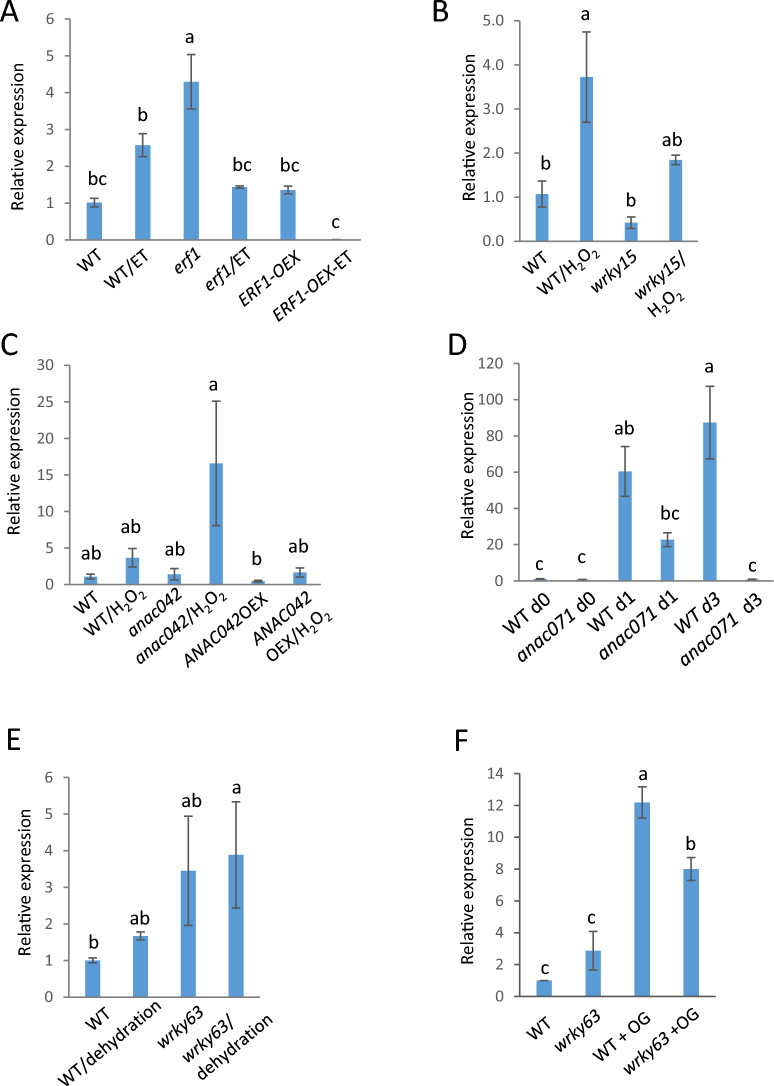
Relative expression of *SAG21* in WT and mutants grown under optimal conditions compared to stress or hormone treated (in brackets for each line): (**A**) *erf1-1* seedlings (treated with 100 ppm ethylene), (**B**) *nac042/JUB1* and *NAC042*OEX seedlings (H_2_O_2_ stress for 6 h before harvest), (**C**) *anac071* stems cut and left on the plant for 0, 1 and 3 days, (**D**) *wrky63* seedlings treated with OG (for 1 h before harvest), (**E**) *wrky63* seedlings (ambient dehydration stress for 30 min after harvest), (**F**) *wrky15 amiRNA* seedlings (H_2_O_2_ stress for 6 h before harvest); mean relative expression ± S.E.; n = 3; different letters indicate significant differences P < 0.05).

To test whether Y1H binding was indicative of a functional role for the TFs in regulating *SAG21* expression *in planta,* expression of *SAG21* was analysed in Arabidopsis mutants of five of the TFs identified in the Y1H screens. Plants were grown under optimal conditions or were exposed to stresses or stress-related plant growth regulators already known to affect *SAG21* expression^24^. Expression of *SAG21* in *ERF1* mutants over-expressor lines was assessed with and without ethylene as this TF is known to mediate responses to ethylene^42^. Under optimal conditions, *SAG21* expression was higher in the *erf1* mutant seedlings than in WT Arabidopsis, but *SAG21* expression was not affected by *ERF1* over-expression (Fig. 5A). Expression of *SAG21* in WT seedlings was up-regulated by exposure to ethylene, however in the *erf1* mutant background this increase was abolished, and *SAG21* expression was in fact down-regulated in response to ethylene compared to untreated controls (Fig. 5A). In the *ERF1* over-expressor (OEX) background seedlings, ethylene treatment again down regulated *SAG21* expression.

Both *ANAC042* (*JUB1*^36^) and *WRKY15*^37^ respond to H_2_O_2_ and could be mediating the response of *SAG21* to ROS. *ANAC042* was also identified as a potential regulator of *SAG21* during senescence (Fig. 4), which in turn might be mediated by ROS. Exposure of WT seedlings to H_2_O_2_ confirmed elevated *SAG21* expression levels (Fig. 5B,C^24^) although the change was not always statistically significant. In seedlings where *ANAC042* expression was reduced, this increase was greatly enhanced, although due to variability the change was again not significant (P < 0.05). In amiRNA *wrky15* seedlings where *WRKY15* expression is reduced, the increase in *SAG21* expression elicited by H_2_O_2_ treatment was slightly (though not significantly) reduced (Fig. 5B). In agreement with the protoplast co-expression experiments, under optimal growth conditions down-regulation of *WRKY15* did not greatly affect *SAG21* expression (Fig. 5B). Likewise, down-regulation of *ANAC042* in the *anac042* (*jub1-1*) plants did not affect expression of *SAG21* under non-stressed conditions (Fig. 5C). However, over-expression of *ANAC042* from the 35S promoter appeared to slightly reduce the expression of *SAG21* under optimal conditions, and slightly dampened upregulation (although again not significantly, probably due to the variability in expression) (Fig. 5C).

Although *ANAC071* was identified as a potential regulator of *SAG21* under drought and pathogen stress (Fig. 4), its most important role recognised to date has been in stem wound responses^43,44^. Expression of *SAG21* increased progressively over time when bolting stems were excised, and stored at ambient temperature, hence wounded, as well as exposed to ambient dehydration (Fig. 5D). Although an increase in *SAG21* expression over time was also seen in *anac071* excised stems after one day of storage, it was abolished after 3 days.

*WRKY63* (also known as *ABO3*) is important in drought and ABA signalling^40^, and also responds to salicylic acid (SA)^15^. Since *SAG21* expression is also upregulated by SA and pathogens^24^ as well as oligogalacturonide (OG) elicitors^32^, the effect of OG elicitors on expression of *SAG21* was tested in *wrky63* insertion mutant seedlings under non-stressed and ambient dehydration stress treatment and in response to OG treatment. Dehydration stress slightly increased *SAG21* expression in seedlings, although not significantly (Fig. 5E). Under optimal conditions, expression of *SAG21* in *wrky63* mutants was elevated compared to WT (although with substantial variability across replicates), in agreement with the co-expression in protoplasts. A more dramatic effect of WRKY63 on *SAG21* expression was seen when *wrky63* mutant seedlings were challenged with OG elicitor (Fig. 5F). In the WT there was a dramatic upregulation of *SAG21* expression which was significantly reduced, though not abolished in *wrky63* mutant seedlings.

## Discussion

The large number of TFs predicted to bind to, and identified as binding to, the *SAG21* promoter indicates a complex regulatory network upstream of this gene. However, there was not complete agreement between TF families predicted to bind, likely TF regulators from co-expression analyses and those discovered by Y1H or found in ChIP databases. This suggests that cryptic TF binding sites are present which are not identified by the tools available such as PlantPan3.0, and, of course, the wide Y1H screen using pools of TFs may easily have missed interactors. However, overall, 33 potential regulators of *SAG21* expression were identified here using the Y1H approach and importantly, confirmed by repeating the Y1H. These included TFs from nine families: 13 WRKY TFs, five ERF and five HB TFs, four NACs, two TCP and one each of ABF, ZAT, HMG-box and MYB. Of these, four WRKYs were further tested in protoplast co-expression assays. Only WRKY63 was able to activate SAG21:GUS reporter expression. This may reflect the limitation of this approach as only leaf mesophyll cells are represented, and other TFs required for activation of *SAG21* promoter activity may not be represented. The functional role of five TFs (one ERF, two WRKY and two NAC family) were tested based on the availability of mutant lines and provide a complex picture of SAG21 promoter activation.

Many ERF transcription factors are involved in the response to abiotic stresses^9^ and may be mediators of stress signals to *SAG21*. Here we showed that five ERF TFs were able to bind to regions of the *SAG21* promoter in a Y1H assay and may be involved in regulating the response of *SAG21* to pathogens. ERF5 is a positive regulator of JA mediated defence and a negative regulator of SA signalling^45^ and ERF14 is also involved in pathogen responses^46^. ERF10 is a repressor up-regulated in response to SA + JA^47^ so that may implicate it in pathogen responses, although it was also upregulated during leaf senescence^48^. ERF15 is a positive regulator of ABA responses^49^ and likely mediates both salinity and drought responses. ERF15 may share a binding site with ERF1 as it also bound the − 1 to − 333 promoter fragment, while the other three ERFs bound the promoter further upstream. The lack of consistency between ERFs co-expressed with *SAG21*, identified through the Y1H screen, and identified though ChIP studies as potential direct regulators of *SAG21* likely reflects the complexity of *SAG21* regulation given that *ERF1* regulation may only be important under specific stress conditions or developmental stages. Other ERFs identified in the ChIP studies (AP2 in inflorescences, ERF115 in roots and BBM in seedlings) may require plant factors to bind to the *SAG21* promoter or may have been missed in the wide TF screen. It has been suggested that some TFs are not always processed correctly in yeast^50^ possibly resulting in false-negatives during the Y1H assay, causing potentially significant TF interactions to be missed.

ERF1 was selected for further study as it plays several roles in both stress responses and development. For example, in plant pathogen interactions ERF1 integrates JA and ET signalling^42^ as it responds synergistically to the two hormones. ERF1 also responds to salinity, heat and drought stresses^30^. In development it mediates ET-induced repression of root growth^51^ and has a role in regulating flowering time^52^. *SAG21* was upregulated in 35S::ERF1 plants under drought stress^30^ and indeed the *SAG21* promoter contains a single GCC element (GCCGCC) in inverse orientation 70 bp upstream from the ATG start codon. This is included in Fragment 1 which was one of the two *SAG21* promoter fragments that bound ERF1 in the Y1H screen. The finding that *SAG21* expression increases in WT Arabidopsis when challenged with ethylene is thus consistent. However, the upregulation of *SAG21* expression in *erf1* mutants in the absence of ethylene suggests that ERF1 may also be acting as a repressor of *SAG21* expression when ethylene is not present. ERF1 was previously reported as a repressor of *FLOWERINGLOCUS T (FT)* to delay floral initiation^51^, so a repression of *SAG21* is not inconsistent with its known functions. The lack of upregulation in ERF1-OEX seedlings under non-stressed conditions is also consistent with the data from Cheng et al.^30^. The higher expression of *SAG21* in *erf1* mutants may reflect other signalling pathways or regulatory components, but the lack of upregulation of *SAG21* in the *erf1* mutant suggests that the upregulation of *SAG21* by ethylene may be mediated primarily through ERF1.

The two TCP TFs confirmed as binding to the *SAG21* promoter (TCP4 and TCP8) may regulate its expression both during development and in response to stress. TCP4 regulates leaf development^53^ and represses petal greening^54^ although it is also involved in the repression of leaf area under high temperature^55^ so has a role in mediating abiotic stress signals. TCP8 is involved in SA signalling as part of host immunity^56^ and also in modulating brassinosteroid responses^57^. Although the *SAG21* promoter was not identified in a ChIP experiment with TCP8, *SAG21* was down-regulated in *tcp8* mutants^57^, showing that there is an effect of TCP8 on *SAG21* expression. The Y1H data here suggest that the interaction could be direct and might have been missed in the ChIP screen.

Of the five homeodomain leucine zipper TFs identified in the initial Y1H screen (Supplementary Table 3) and confirmed as Y1H interactors, HB5 was identified in a ChIP experiment with ABA treated seedlings and indeed HB5 is a likely ABA signalling regulator in seedlings^58^. HB12 is involved in root and leaf development under non-stressed conditions, and seed production under drought conditions^59^. HB13 is involved in seed to seedling development^60^ but also in drought and salinity tolerance^61^. HB23 is involved in co-regulating blue light signalling and growth^62^. HB52 is also involved in light responses, and their integration with nitrogen status^63^, but is also ethylene-responsive and controls primary root elongation^64^. These transcription factors may therefore be important in regulating *SAG21* during plant growth while responding to environmental signals and are worthy of further investigation.

Of the other four TFs identified in the initial 1500 TF Y1H screen, and confirmed as Y1H interactors, ABF4 was also identified as a potential upstream regulator of *SAG21* in the hCSI modelling during pathogen attack. ABF4 is involved in ABA signalling^65^ and ABA was discovered to be a negative regulator of *B. cinerea* infection^66^. Of the remaining three TFs confirmed as Y1H interactors with the *SAG21* promoter from the initial screen, ZAT10/STZ is induced by a wide range of abiotic stresses including salt, cold, ABA and dehydration^67^ and was also recently identified as a hub gene in plant pathogen interactions^68^ and MYB67 is highly upregulated during seed dormancy^69^. These TFs may therefore mediate some of the upregulation of *SAG21* expression elicited by ABA^27^. Finally, AT1G76110 is part of the light response network^70^ and therefore may be involved in the known light regulation of SAG21^24,27^.

NAC TFs play important roles in mediating stress responses^8^. Two NAC TFs were identified from the ChIP seq database data as binding to the *SAG21* promoter: *ANAC032* and *ANAC102* in ABA challenged seedlings (Supplementary Table 2^71^) while six other NAC TFs were co-expressed with *SAG21* (Supplementary Table 1): these include *ANAC033*, *ANAC70* and *ANAC100* under environmental stress conditions and *ANAC81, ANAC87* and *ANAC91* under hormone treatments^8^. Of these only *ANAC102* was not included in the Y1H screen. Four different NAC TFs from those co-expressed or detected in ChIP experiments (ANAC013, ANAC038/39, ANAC042 and ANAC071) were confirmed to bind to the *SAG21* promoter in the − 1 to − 133 region (Fragment 1) by Y1H. However, no canonical motifs for NAC TFs, i.e., CGT[G/A]^35^ were found in this region of the *SAG21* promoter. This binding may be explained because the CGT[G/A] canonical sequence^72^ is seemingly not universal across all NAC TFs. For example, ANAC042 which binds to the *SAG21* promoter, bound to a different core sequence RRYGCCGT^36^. Since mutation of the GCCGT sequence in the *SAG21* promoter did not abolish binding of any of the four NAC TFs, there must be other cryptic sites in this sequence that enable binding of these NAC TFs. ANAC038/39 was identified in the hCSI modelling as a potential regulator of *SAG21* expression during *Botrytis* and *Pseudomonas* infection, and bound to Fragment 1 of the *SAG21* promoter. Expression of this NAC TF is upregulated by *Botrytis* infection^73^ as is *SAG21*^24^ so this role would warrant further verification.

The functional role of two of the NAC TFs identified in the *SAG21* promoter Y1H screen was assessed. *ANAC042,* also known as JUB1, is a negative regulator of leaf senescence and is strongly upregulated by H_2_O_2_^36^. The increased upregulation of *SAG21* expression in the *anac042* mutant background when treated with H_2_O_2_ suggests that *ANAC042* may be acting as a negative regulator of *SAG21* expression by ROS. This is consistent with the role of ANAC042 as a repressor in the regulation of anthocyanin biosynthetic genes^36^. ANAC042 is also important as a negative regulator of senescence^36^ and our modelling suggested that this TF might be a regulator of *SAG21* during this stage of development. *SAG21* is only very transiently expressed during developmental senescence^24,28^. Nevertheless, over-expression of *SAG21* delayed leaf senescence, so it may play a role in mitigating the effects of rising ROS during senescence.

**Figure 6:**
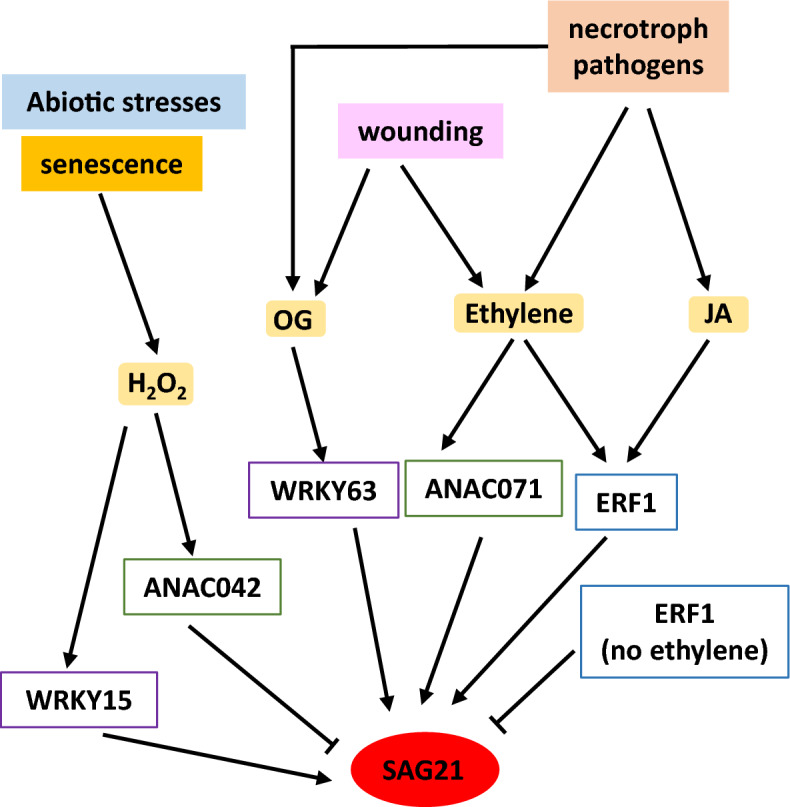
Model of upstream regulators of *SAG21* expression based on the Y1H screens and real time qPCR. Biotic and abiotic stress signals activate plant hormones and signalling molecules which in turn activate TFs belonging to several families. These TFs activate and repress *SAG21* expression depending on the condition.

A much clearer effect on *SAG21* expression was detected in *anac071* mutants where wound-induced *SAG21* upregulation was significantly impaired (Fig. 5D). *ANAC071* is involved in wounding repair: its expression is activated 1–3 days after wounding in Arabidopsis stems and *anac071* mutants show impaired vascular tissue regeneration following wounding^43^. The function of *SAG21* in wound repair is unclear, but it is strongly and rapidly (within minutes) upregulated by wounding also in leaves^24^. This long-term upregulation of *SAG21*expression 3 days after wounding suggests that *SAG21* may have a role both in early and later wounding responses. Wounding elicits the production of ethylene within 20 min of wounding^74^ and *ANAC071* upregulation around wounding sites is thought to be regulated by both auxin accumulation and ethylene. As *SAG21* expression is also ethylene induced, this might be part of the signalling pathway (Fig. 6). However, *SAG21* expression following wounding may also be induced by OGs via an increase in H_2_O_2._ Wounding elicits a rise in H_2_O_2_ activated by OG release from the damaged cells^75^. H_2_O_2_ accumulates gradually at the site of wounding^76^ acting as a signalling molecule to activate defence responses, and hence could activate *SAG21* expression, since *SAG21* expression is induced by ROS^24,27^.

As well as NACs, WRKY TFs are another major plant TF family involved in the regulation of both biotic and abiotic stress signals^6,8^. Eight WRKY TF genes were co-expressed with *SAG21*, including *WRKY15* that was co-expressed under environmental and biotic stress as well as hormone treatment (Supplementary Table 3). Only two of these TFs, WRKY15 and WRKY33, bound to the *SAG21* promoter in the Y1H screen. Although activation of *SAG21* expression by either WRKY15 or WRKY33 was not confirmed in protoplast co-expression assays, induction of *SAG21* expression by H_2_O_2_ was reduced (though not significantly) in the *wrky15* mutant indicating that WRKY15 may be contributing to the upregulation of *SAG21* expression by H_2_O_2._ As well as these two WRKY TFs, a further eleven were identified as binding to the *SAG21* promoter through Y1H analysis. All 13 WRKY TFs identified in the screen bound to Fragment 2 (Fig. 1) but only two of these bound to Fragment 1. As there is a large overlap of 155 bp between these two fragments, this suggests that the region from − 333 to − 479 bp upstream of the ATG is playing a major role in WRKY TF recognition. Indeed, all these WRKY TFs appeared to bind to W-box 5 (located at − 362) but not W-box 4 (located at − 324) apart from WRKY33 that binds to both cis-elements. In addition, WRKY63 and WRKY67 also bind to some other cryptic site(s) within Fragment 2 of the *SAG21* promoter and were the only two WRKY TFs that bound to more than one promoter fragment, interacting with a total of four separate fragments of the 1685 bp *SAG21* promoter. Different WRKY TFs have differing specificities for variants of the W-box^11^ and binding of WRKY TFs to anomalous sites has also been reported^77,78^. For example, WRKY70 can bind to the YGACTTTT sequence^77^ and this sequence is also present at positions − 38 and − 368 in the *SAG21* promoter, found in Fragment 1 and Fragment 2, respectively. However, regardless of the presence of this motif in both fragments, WRKY70 was not identified in our Y1H screens. WRKY63 and WRKY67 are phylogenetically similar and are members of the IIIa sub-group^15^ that share a highly conserved amino acid region outside of the WRKY domain, hence may also share a yet undiscovered alternative binding sequence within target promoters.

Co-expression studies of the SAG21p-GUS reporter construct and 35S-WRKY candidate genes in protoplasts confirmed the activation of *SAG21* expression by WRKY63. *WRKY63* also known as *ABO3* is important in drought and ABA signalling^40^. However, *SAG21* expression was higher under both non-stress and dehydration stress treatments in the *wrky63* mutant background (although with increased variability) suggesting that it might actually be acting as a repressor. WRKY63 can act as a repressor of MYB28/29 under water deficit^79^ hence this role is plausible. *WRKY63* mutation had a stronger effect on the response of *SAG21* to the OG elicitor. Upregulation of *SAG21* expression by OG has been previously reported^32^ and is consistent with its upregulation by both fungal and bacterial pathogens^24^ independently of a hypersensitive response. *SAG21* expression is also upregulated in response to flg22 a peptide elicitor derived from bacterial flagellin^32,80^. *WRKY63* expression responds to SA and is amongst the IIIa sub-group WRKY genes that require SA accumulation to induce their expression following infection with *Pernospora parasitica*^15^. Surprisingly, the study by Denoux et al.^32^ did not show that WRKY63 expression responded to OGs. The partial dependence of *SAG21* upregulation on *WRKY63* in response to the OG elicitor may therefore be mediated by SA induction of *WRKY63*. Of note is that WRKY63 is involved in the activation of BCS1, a mitochondrial protein, in response to flg22 treatment^14^ and the expression pattern of *WRKY63* indicated a role as a positive regulator of mitochondrial proteins upon stress. Since SAG21 is a mitochondrial protein that also responds to ROS, the role of WRKY63 as an activator is consistent. The contrasting results on the role of WRKY63 on *SAG21* expression likely reflect different roles of this TF under different conditions.

The lack of *SAG21* promoter activation in protoplasts when co-expressed with WRKY15, WRKY33 and WRKY67 could indicate that the Y1H binding to the *SAG21* promoter is not functionally significant or that activation only occurs under specific stresses or developmental stages. A role of WRKY15 in regulating the response of *SAG21* to ROS would be consistent with its role in mediating communication between mitochondrial stress responses^37^, as well as WRKY33, a key regulator of pathogen responses^38^, and WRKY67 which is activated by salt stress^41^. Other WRKY TFs binding to the *SAG21* promoter are also worthy of further investigation through the analysis of *SAG21* expression in mutant lines.

In conclusion, three TFs belonging to three different families have been identified as regulators of *SAG21* expression, ERF1, ANAC071 and WRKY63. Their functional role has been confirmed through analysis of Arabidopsis TF mutant lines. A further two TFs: WRKY15 and ANAC042 may also regulate *SAG21* expression, although further verification is needed. A tentative model of their interaction is proposed (Fig. 6). However, further work will be needed to assess the functional significance of other TF interactions with *SAG21* identified here through Y1H screens. Analysing *SAG21* expression under different developmental stages with and without different stresses and/or stress combinations, in combination with ChIP analysis of candidate TFs and a detailed analysis of the *SAG21* promoter is essential to identify crucial promoter elements involved in its transcriptional regulation. This will enable the regulatory network controlling this complex promoter to be disentangled from the bottom up and top down.

## Methods

### Analysis of transcription factor binding sites and cloning of promoter fragments

PlantPAN3.0^81^ was used to identify TFs that co-express with *SAG21/AtLEA5/LEA38* (At4g02380) and ChIP experiments showing binding of TFs to 1685 bp upstream of the *SAG21* ATG start codon.

PlantPAN3.0^81^ was also used to find predicted cis-elements in this 1685 bp region. The 1685 bp was divided into seven overlapping fragments of approximately 300 bp based on this analysis for use in Y1H screens. Fragments were amplified using gene-specific primers (Supplementary Table 4) and cloned into the pHISLEU2 vector (Invitrogen) using restriction enzymes *Eco*RI and *Mlu*I. All cloned fragments were sequenced (deposited to Genbank with accession codes OR513440–OR513446) and then transformed into the Y187 (MATα) yeast strain (Clontech) to form baits for the Y1H screens.

### Y1H screens

Three libraries of TFs were screened containing full-length TF open reading frames fused to the N-terminal GAL4 activation domain in the vector pDEST22 (Invitrogen). The first library comprised approximately 1500 TFs (REGIA + REGULATORS; RR Library^82^ and was kindly donated by the authors. The TF library was transformed into the yeast strain AH109 (MATa) and pooled as in Ref.^50^. The other two libraries comprised 94 NAC TFs and 75 WRKY TFs, and consisted of sequence verified constructs (Supplementary Table 5^50^).

The libraries were screened essentially as in Ref.^50^. Yeast clones were grown on SD-Trp (library) and SD-Leu (bait) and mated on YPDA medium, then replica plated onto SD-Leu-Trp, SD-Leu-Trp-His with the addition of 1–100 mM 3-amino-1,2,4-triazol (3AT). Successful mating was checked by growth on SD-Leu-Trp. After overnight incubation and replica cleaning, plates were incubated for 4 days. Positive colonies (growing on SD-Leu-Trp-His medium) were patched onto selective plates. Multiple yeast clones of each promoter fragment were tested against the libraries to ensure consistent results. To identify the interacting TF from the 1500 TF library, yeast colonies were treated with 20 mM NaOH at 100 °C for 10 min, and then subjected to nested colony PCR. The first amplification was with primers SABR447 and SABR448; for the second amplification, PCR product was diluted 10×  and amplified with primers SABR150 and SABR4506 (primer sequences in Supplementary Table 4). PCR products from the second PCR were identified by partial sequencing following purification with a QIAquick kit (Qiagen). TFs were identified using WuBLAST within TAIR. Screening of NAC and WRKY TF libraries was carried out in the same way except sequencing of positive colonies was not required.

To verify the Y1H positive TF interactions, plasmids carrying individual TFs were transformed into the Y187 yeast strain and mated with the bait-carrying yeast strain as above. Three independent yeast transformants for each promoter fragment bait construct were tested.

### Mutation of cis-elements in promoter fragments

W-box sequences and the NAC binding sequence (TGCC[GT]^36^) were mutated using a QuikChange Lightning Site-Directed Mutagenesis Kit (Agilent Technologies). Primers are listed in Supplementary Table 4. Mutated clones were sequenced (deposited to Genbank with accession codes OR513447–OR513450), and a second round of mutagenesis was applied to introduce mutations into both W-box sequences within the same promoter fragment. For each Y1H assay, four separate promoter clones (containing the mutation) were used, along with a non-mutated promoter fragment clone as a positive control.

### Analysis of transcription factor binding in protoplasts

Protoplasts were isolated from Arabidopsis leaves and transformed transiently essentially as in Ref.^83^. Protoplast integrity and number were checked using a light microscope. Protoplasts were co-transformed With both pGPTV-Kan-SAG21(1685)::GUS (as described in Ref.^24^) and control plasmid pJIT60-35S::LUC or TF plasmids pJIT60-35S::WRKY15, pJIT60-35S::WRKY63, or pJIT60-35S::WRKY67. All the pJIT60-35S clones were created from pDONRZeo entry clones via Gateway cloning (Invitrogen). Clones were sequenced to verify integrity of the TF coding sequences. A luciferase assay kit (Promega) was used to detect LUC and a methyl-umbelliferone (MUG) based β-glucuronidase assay for detection of GUS, and both reporters were analysed with a TECAN M2000 pro Fluorimeter. pJIT60-35S::GAL4DB was used as a negative control instead of the TF clone. LUC luminescence was used to verify transformation, GUS fluorescence was used to assess promoter activation. Assays were repeated three times. Data were normalised against LUC and then presented as relative expression to the GALDB control.

### hCSI modelling

Hierarchical Causal Structure Inference (hCSI) analysis^84^ was based on the detailed time course microarray data sets generated under the PRESTA project following leaf senescence over 3 weeks^85^, infection with *Botrytis cinerea* over 48 h^73^, *Pseudomonas syringae* infection over 17.5 h^86^ and 6 h of high light treatment^87^.

### Plant material and growth

All *Arabidopsis thaliana* genotypes were in the Columbia background. A homozygous *erf1* knock out (KO) line was selected from segregating lines containing a T-DNA insertion (GK-850A03/N481507) obtained from NASC (Nottingham Arabidopsis Stock Centre). *ERF1* over-expressor (OEX) 35S::ERF1-1 (AT3G23240/N6143) was also obtained from NASC. A homozygous artificial miRNA (amiRNA) line for *WRKY15* (AT2G23320) that knocks down *WRKY15* expression was as described in Ref.^37^. T-DNA insertion lines for *wrky63* (At1g66600; SALK_068280C) were obtained from NASC. Insertion position in the knockouts were verified by PCR (Supplementary Figs. 3–6), insertion knocks out expression of *WRKY63*^40^. *Anac071* (SALK_012841C) seeds were as in Ref.^43^. A T-DNA insertion line of a*nac042* (also known as *jub1-1;* AT2G43000; SALK_036474) in which expression was knocked down and of 35S::ANAC042 over-expression line were as in Ref.^36^.

All seeds were surface sterilised and stratified in sterilised distilled water for 24 h at 4 °C. For growth on Petri dishes seeds were sown onto sterile 1× Murashige and Skoog (MS) basal salt mix, 1% agar, 1% sucrose, pH 5.5–5.7 and sealed with MicroporeTM tape. Seedlings were germinated and grown in an environmentally controlled growth chamber at 21 °C under long days (16 h day, 8 h night). For obtaining stem tissue, seeds were sown onto freeze sterilized John Innes soil mix (soil: sand at 3:1 ratio) and grown in a growth chamber (16 h light, 21 °C) until bolting. Following bolting, plants were grown for an additional 7 days, and stem tissue was then excised.

### Stress treatments

Seedlings were pre-treated with 12 h light (90–100 μmol photons m^−2^ s^−2^) before treatment to repress expression of the *SAG21* gene which is light regulated^24,27^. Ethylene (100 ppm) was injected into sealed Petri dishes containing 7 d old seedlings and then left for 24 h in the light. As a dehydration stress treatment 14 d old seedlings were removed from Petri dishes, placed on Whatmann filter paper for 30 min and exposed to air flow in a laminar air flow cabinet. For the control treatment, Petri dishes were left in the growth chamber for 30 min^88^. Hydrogen peroxide stress treatment of 14 d old seedlings was carried out by submerging seedlings in 1× MS liquid medium with 10 mM H_2_O_2_ for 6 h as previously described^36^, control seedlings were left on the plates for the 6 h period. To analyse wound response in stem tissue, stems of soil grown flowering plants were excised just above the rosette leaves, placed into sterile 15 mL falcon tubes and left for 1 or 3 days at room temperature. Stem tissue was then excised from just below the first cauline leaves. To test elicitor responses, seedlings were challenged with a galacturonan oligosaccharide mixture (OG, DP10/DP15elicitor, Elicityl). Whole rosettes of 4-week-old plants grown in soil were vacuum infiltrated in a 200 µM solution of OG for 5 min. After 1 h of treatment, leaves were then snap-frozen in liquid nitrogen. Control leaves were totally immersed in water. Whole seedlings, leaves or stem sections from three biological replicates of the control and stress treatments were harvested and frozen in liquid nitrogen and stored at − 80 °C.

### Analysis of gene expression by real time qPCR

Plant material (100–150 mg) was ground into a fine powder with liquid nitrogen and RNA isolation was carried out using an RNeasy Plant Mini Kit (QIAGEN) or using TRI-Reagent (Sigma). Residual genomic DNA was removed using RQ1 DNase (Promega). First strand cDNA was synthesized from RNA samples using M-MLV RNase H Reverse Transcriptase (Promega) using oligo dT (Promega). Real time qPCR used 60 ng of cDNA, 0.4 μL of *SAG21* forward and reverse primers (10 μM) (Supplementary Table 4), 10 μL of 2× qPCRBIOSyGreen Mix Lo-ROX (PCR Biosystems), in a 20 μL reaction volume, and was conducted using a Light Cycler 96 (Roche) machine. PCR thermal profiling conditions were: 95 °C for 120 s, 95 °C for 30 s, 60 °C for 30 s, 72.0 °C for 30 s for 35 cycles followed by melting curve analysis from 60 to 98 °C to check for primer specificity and primer dimers. Gene expression analysis was carried out using the relative comparative method^89^ using the 2−ΔΔct method.

### Plant materials

Use of plants in the present study complies with international, national and institutional guidelines. No seeds were obtained as collections from wild populations of Arabidopsis.

### Supplementary Information

Supplementary Information.

## Data Availability

All key data are provided in the Supplementary Files. Sequences have been deposited with Genbank (Accession Numbers OR513440–OR513450).
