# Supplementary material for: Expression of the Arabidopsis redox-related LEA protein, SAG21 is regulated by ERF, NAC and WRKY transcription factors
Source: Sci Rep. 2024 Apr 2;14:7756. doi: 10.1038/s41598-024-58161-0 (PMC10987515; doi:10.1038/s41598-024-58161-0)
Supplement: Supplementary file 1 — Supplementary Information. [file 41598_2024_58161_MOESM1_ESM.pptx]

## Slide 1
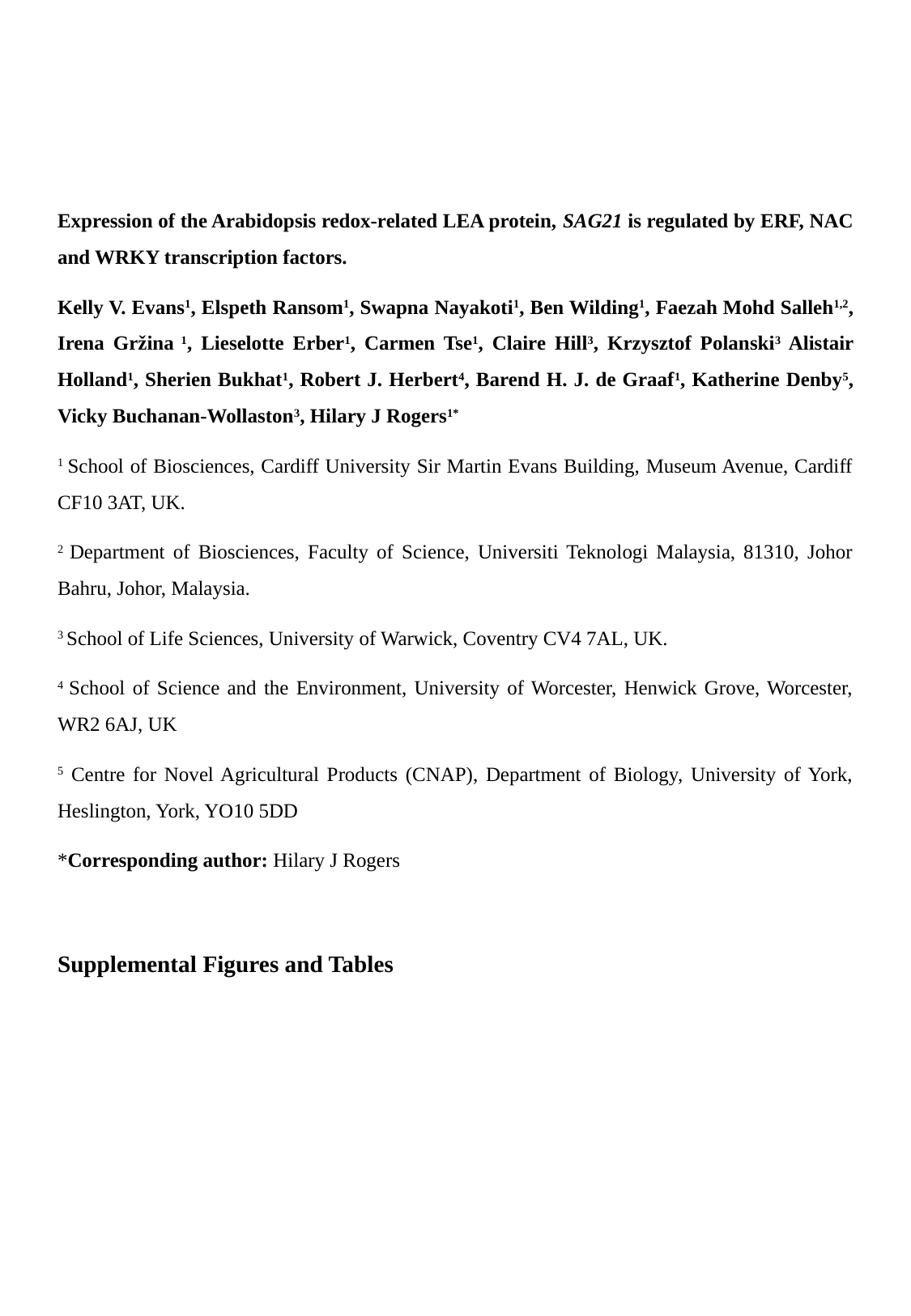

Expression of the Arabidopsis redox-related LEA protein, SAG21 is regulated by ERF, NAC and WRKY transcription factors.
Kelly V. Evans1, Elspeth Ransom1, Swapna Nayakoti1, Ben Wilding1, Faezah Mohd Salleh1,2, Irena Gržina 1, Lieselotte Erber1, Carmen Tse1, Claire Hill3, Krzysztof Polanski3 Alistair Holland1, Sherien Bukhat1, Robert J. Herbert4, Barend H. J. de Graaf1, Katherine Denby5, Vicky Buchanan-Wollaston3, Hilary J Rogers1*
1 School of Biosciences, Cardiff University Sir Martin Evans Building, Museum Avenue, Cardiff CF10 3AT, UK.
2 Department of Biosciences, Faculty of Science, Universiti Teknologi Malaysia, 81310, Johor Bahru, Johor, Malaysia.
3 School of Life Sciences, University of Warwick, Coventry CV4 7AL, UK.
4 School of Science and the Environment, University of Worcester, Henwick Grove, Worcester, WR2 6AJ, UK
5 Centre for Novel Agricultural Products (CNAP), Department of Biology, University of York, Heslington, York, YO10 5DD
*Corresponding author: Hilary J Rogers
Supplemental Figures and Tables

## Slide 2
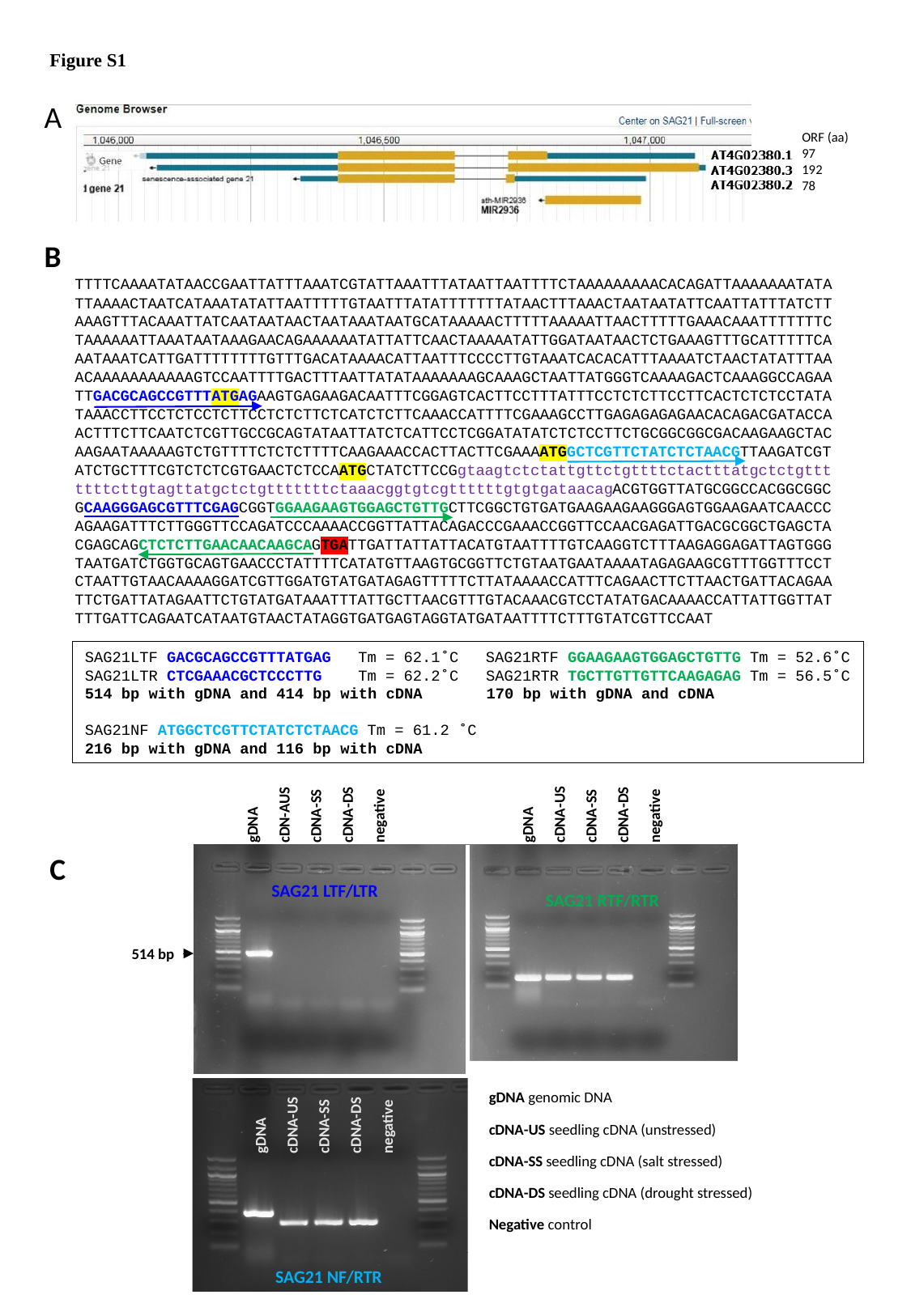

Figure S1
A
ORF (aa)
97
192
78
B
SAG21LTF GACGCAGCCGTTTATGAG Tm = 62.1˚C SAG21RTF GGAAGAAGTGGAGCTGTTG Tm = 52.6˚C
SAG21LTR CTCGAAACGCTCCCTTG Tm = 62.2˚C SAG21RTR TGCTTGTTGTTCAAGAGAG Tm = 56.5˚C
514 bp with gDNA and 414 bp with cDNA 170 bp with gDNA and cDNA
SAG21NF ATGGCTCGTTCTATCTCTAACG Tm = 61.2 ˚C
216 bp with gDNA and 116 bp with cDNA
gDNA
cDNA-US
cDNA-SS
cDNA-DS
negative
gDNA
cDN-AUS
cDNA-SS
cDNA-DS
negative
C
SAG21 LTF/LTR
SAG21 RTF/RTR
514 bp
gDNA
cDNA-US
cDNA-SS
cDNA-DS
negative
gDNA genomic DNA
cDNA-US seedling cDNA (unstressed)
cDNA-SS seedling cDNA (salt stressed)
cDNA-DS seedling cDNA (drought stressed)
Negative control
SAG21 F/R
SAG21 NF/RTR

## Slide 3
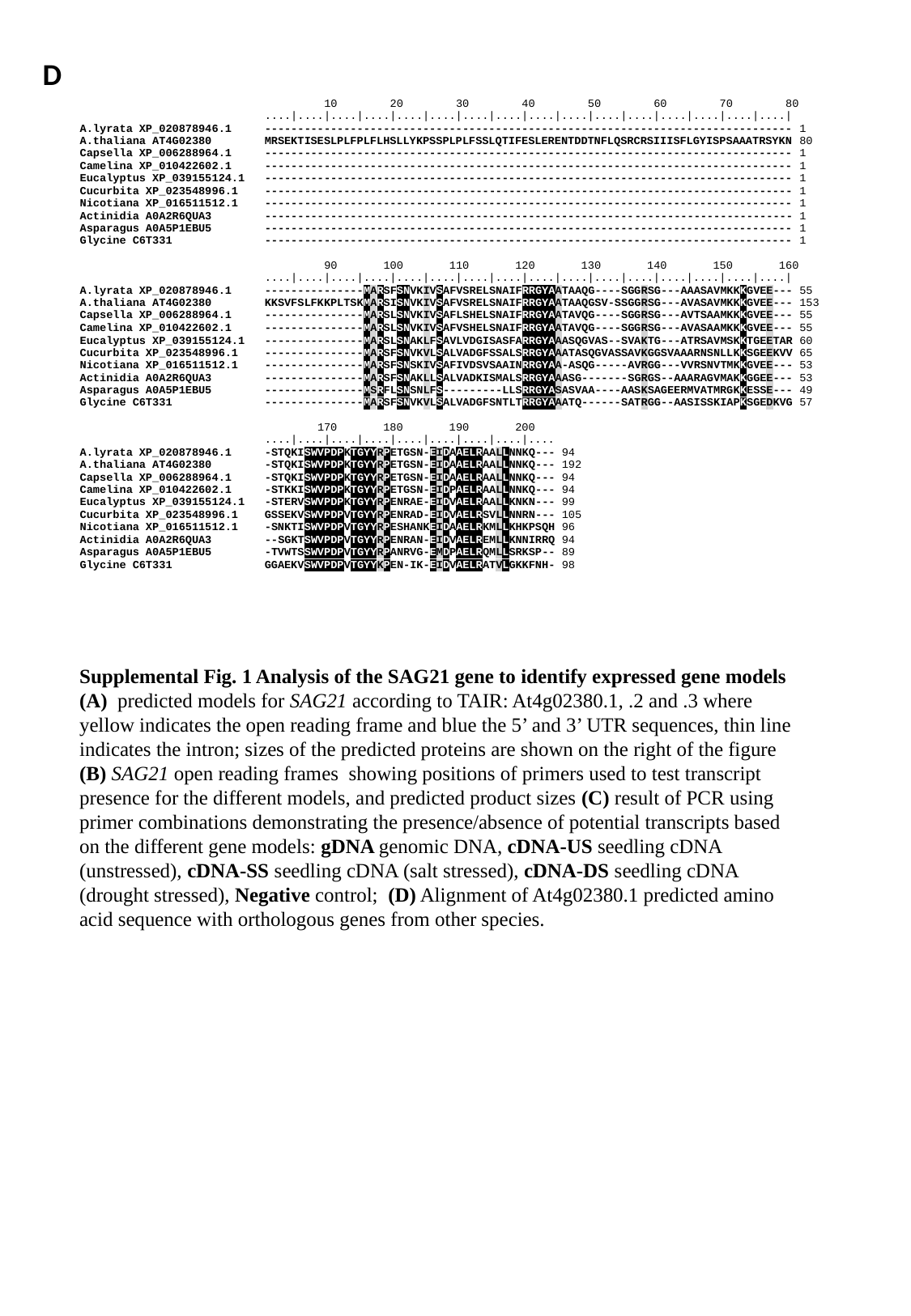

D
Supplemental Fig. 1 Analysis of the SAG21 gene to identify expressed gene models (A) predicted models for SAG21 according to TAIR: At4g02380.1, .2 and .3 where yellow indicates the open reading frame and blue the 5’ and 3’ UTR sequences, thin line indicates the intron; sizes of the predicted proteins are shown on the right of the figure (B) SAG21 open reading frames showing positions of primers used to test transcript presence for the different models, and predicted product sizes (C) result of PCR using primer combinations demonstrating the presence/absence of potential transcripts based on the different gene models: gDNA genomic DNA, cDNA-US seedling cDNA (unstressed), cDNA-SS seedling cDNA (salt stressed), cDNA-DS seedling cDNA (drought stressed), Negative control; (D) Alignment of At4g02380.1 predicted amino acid sequence with orthologous genes from other species.

## Slide 4
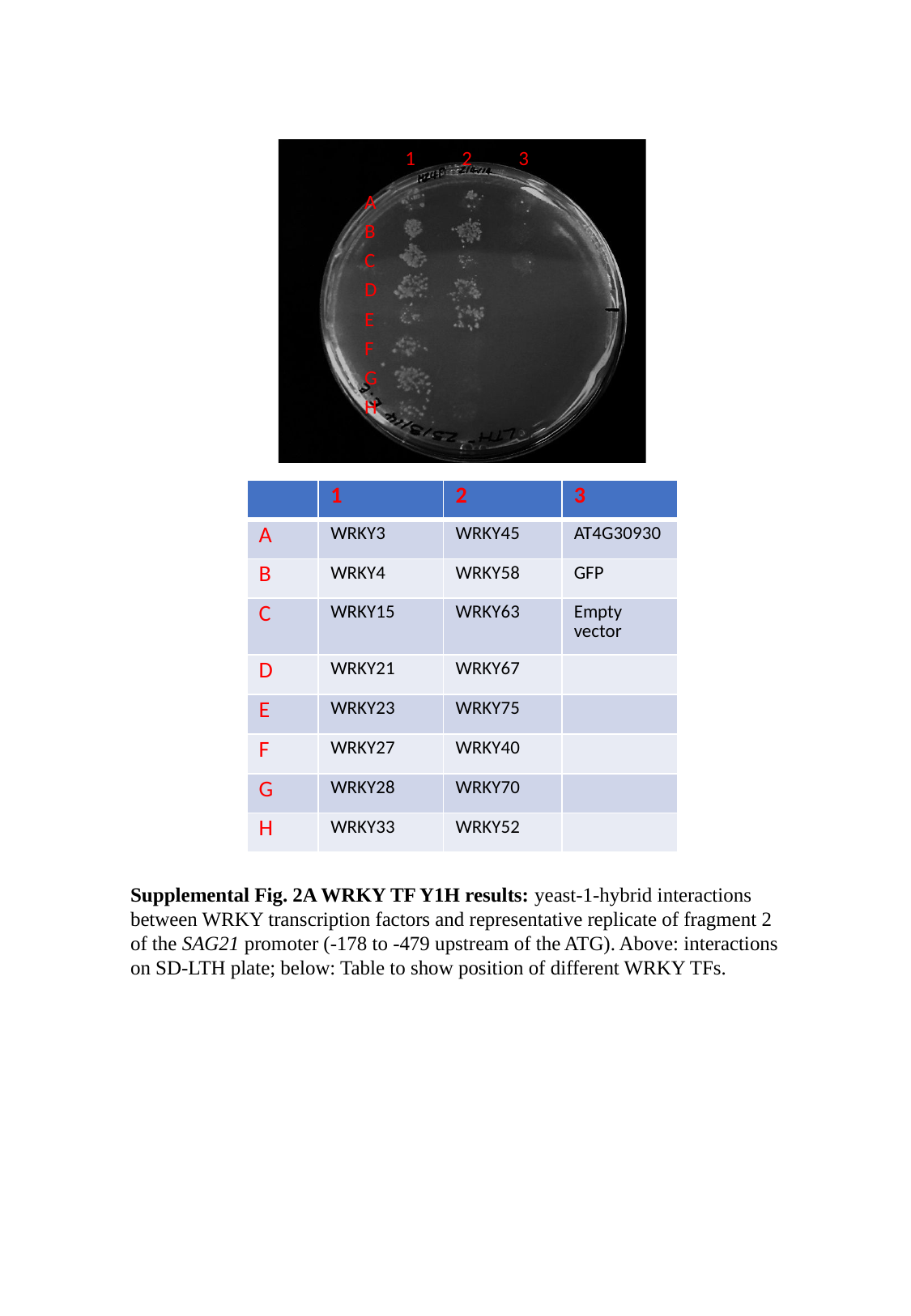

1 2 3
A
B
C
D
E
F
G
H
| | 1 | 2 | 3 |
| --- | --- | --- | --- |
| A | WRKY3 | WRKY45 | AT4G30930 |
| B | WRKY4 | WRKY58 | GFP |
| C | WRKY15 | WRKY63 | Empty vector |
| D | WRKY21 | WRKY67 | |
| E | WRKY23 | WRKY75 | |
| F | WRKY27 | WRKY40 | |
| G | WRKY28 | WRKY70 | |
| H | WRKY33 | WRKY52 | |
Supplemental Fig. 2A WRKY TF Y1H results: yeast-1-hybrid interactions between WRKY transcription factors and representative replicate of fragment 2 of the SAG21 promoter (-178 to -479 upstream of the ATG). Above: interactions on SD-LTH plate; below: Table to show position of different WRKY TFs.

## Slide 5
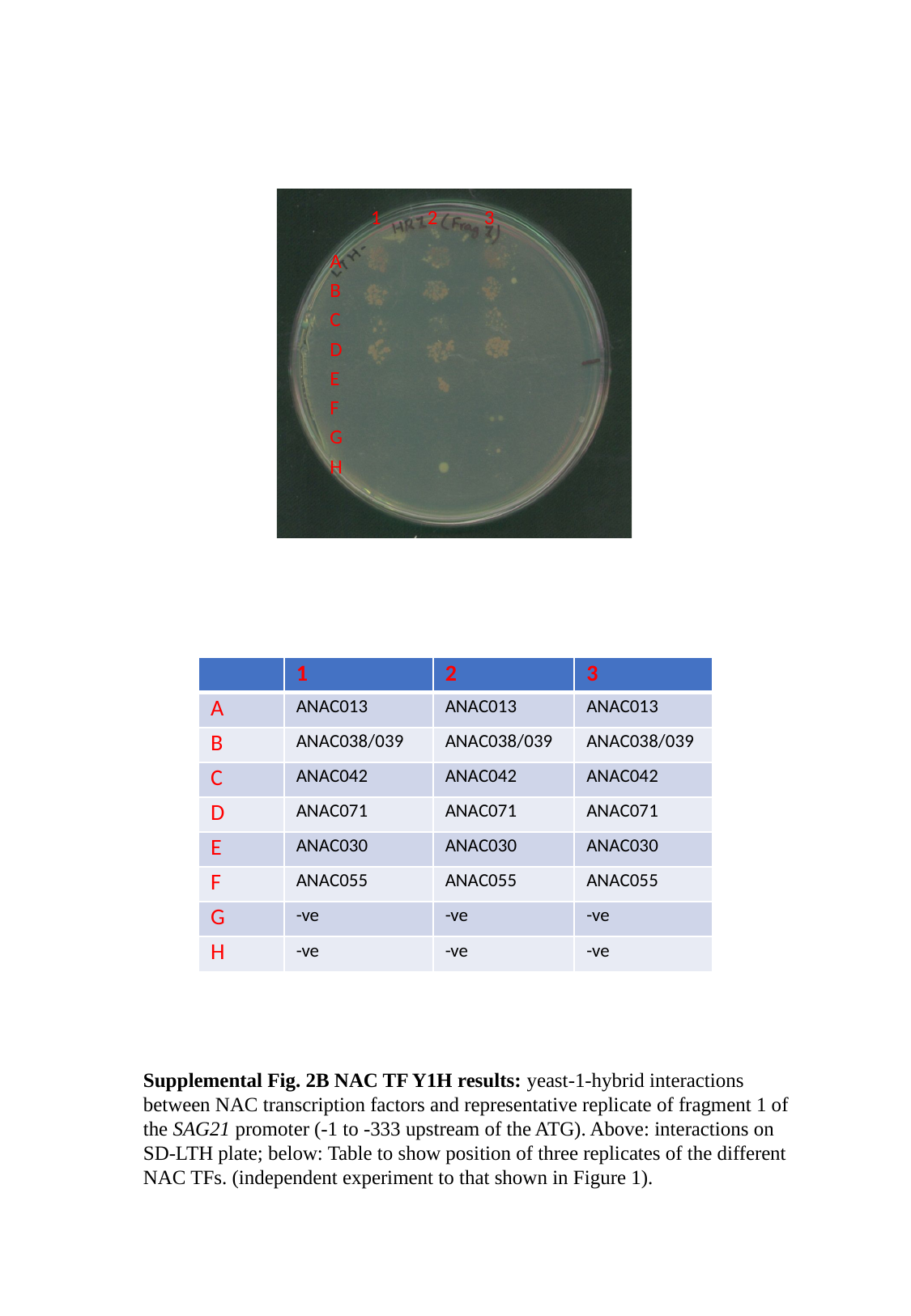

1 2 3
A
B
C
D
E
F
G
H
| | 1 | 2 | 3 |
| --- | --- | --- | --- |
| A | ANAC013 | ANAC013 | ANAC013 |
| B | ANAC038/039 | ANAC038/039 | ANAC038/039 |
| C | ANAC042 | ANAC042 | ANAC042 |
| D | ANAC071 | ANAC071 | ANAC071 |
| E | ANAC030 | ANAC030 | ANAC030 |
| F | ANAC055 | ANAC055 | ANAC055 |
| G | -ve | -ve | -ve |
| H | -ve | -ve | -ve |
Supplemental Fig. 2B NAC TF Y1H results: yeast-1-hybrid interactions between NAC transcription factors and representative replicate of fragment 1 of the SAG21 promoter (-1 to -333 upstream of the ATG). Above: interactions on SD-LTH plate; below: Table to show position of three replicates of the different NAC TFs. (independent experiment to that shown in Figure 1).

## Slide 6
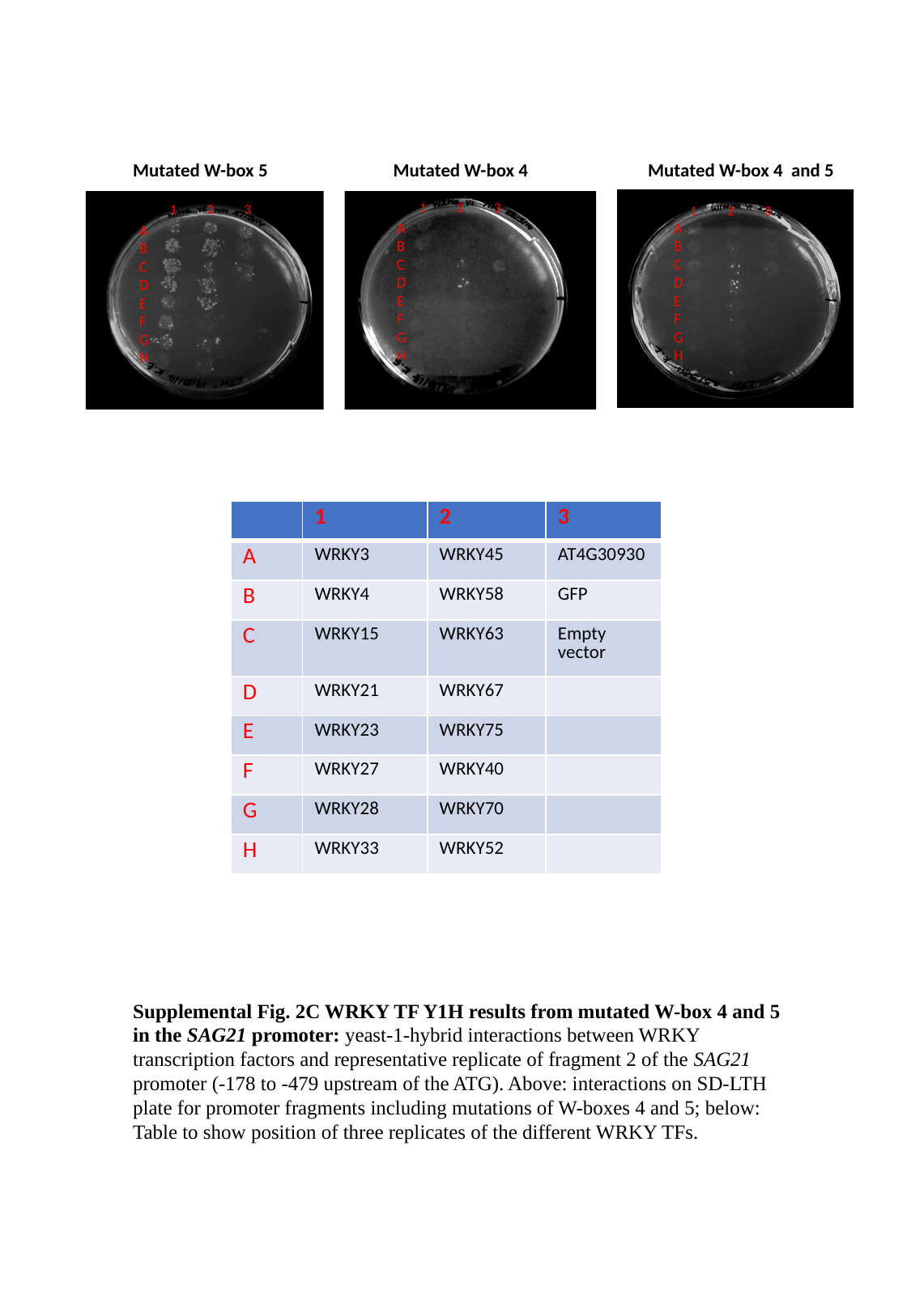

Mutated W-box 5
Mutated W-box 4
Mutated W-box 4 and 5
1 2 3
1 2 3
1 2 3
A
B
C
D
E
F
G
H
A
B
C
D
E
F
G
H
A
B
C
D
E
F
G
H
| | 1 | 2 | 3 |
| --- | --- | --- | --- |
| A | WRKY3 | WRKY45 | AT4G30930 |
| B | WRKY4 | WRKY58 | GFP |
| C | WRKY15 | WRKY63 | Empty vector |
| D | WRKY21 | WRKY67 | |
| E | WRKY23 | WRKY75 | |
| F | WRKY27 | WRKY40 | |
| G | WRKY28 | WRKY70 | |
| H | WRKY33 | WRKY52 | |
Supplemental Fig. 2C WRKY TF Y1H results from mutated W-box 4 and 5 in the SAG21 promoter: yeast-1-hybrid interactions between WRKY transcription factors and representative replicate of fragment 2 of the SAG21 promoter (-178 to -479 upstream of the ATG). Above: interactions on SD-LTH plate for promoter fragments including mutations of W-boxes 4 and 5; below: Table to show position of three replicates of the different WRKY TFs.

## Slide 7
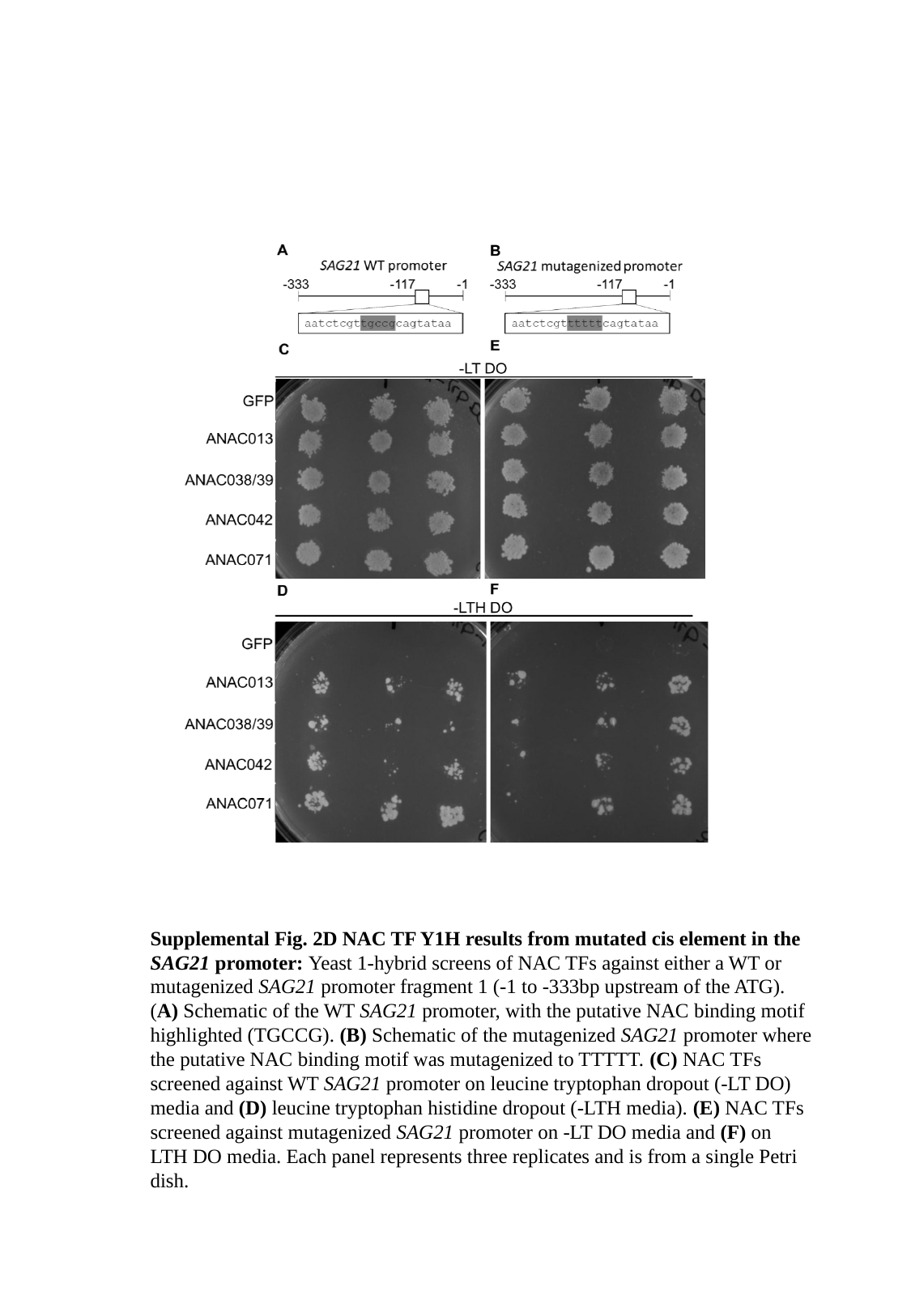

Supplemental Fig. 2D NAC TF Y1H results from mutated cis element in the SAG21 promoter: Yeast 1-hybrid screens of NAC TFs against either a WT or mutagenized SAG21 promoter fragment 1 (-1 to -333bp upstream of the ATG). (A) Schematic of the WT SAG21 promoter, with the putative NAC binding motif highlighted (TGCCG). (B) Schematic of the mutagenized SAG21 promoter where the putative NAC binding motif was mutagenized to TTTTT. (C) NAC TFs screened against WT SAG21 promoter on leucine tryptophan dropout (-LT DO) media and (D) leucine tryptophan histidine dropout (-LTH media). (E) NAC TFs screened against mutagenized SAG21 promoter on -LT DO media and (F) on LTH DO media. Each panel represents three replicates and is from a single Petri dish.

## Slide 8
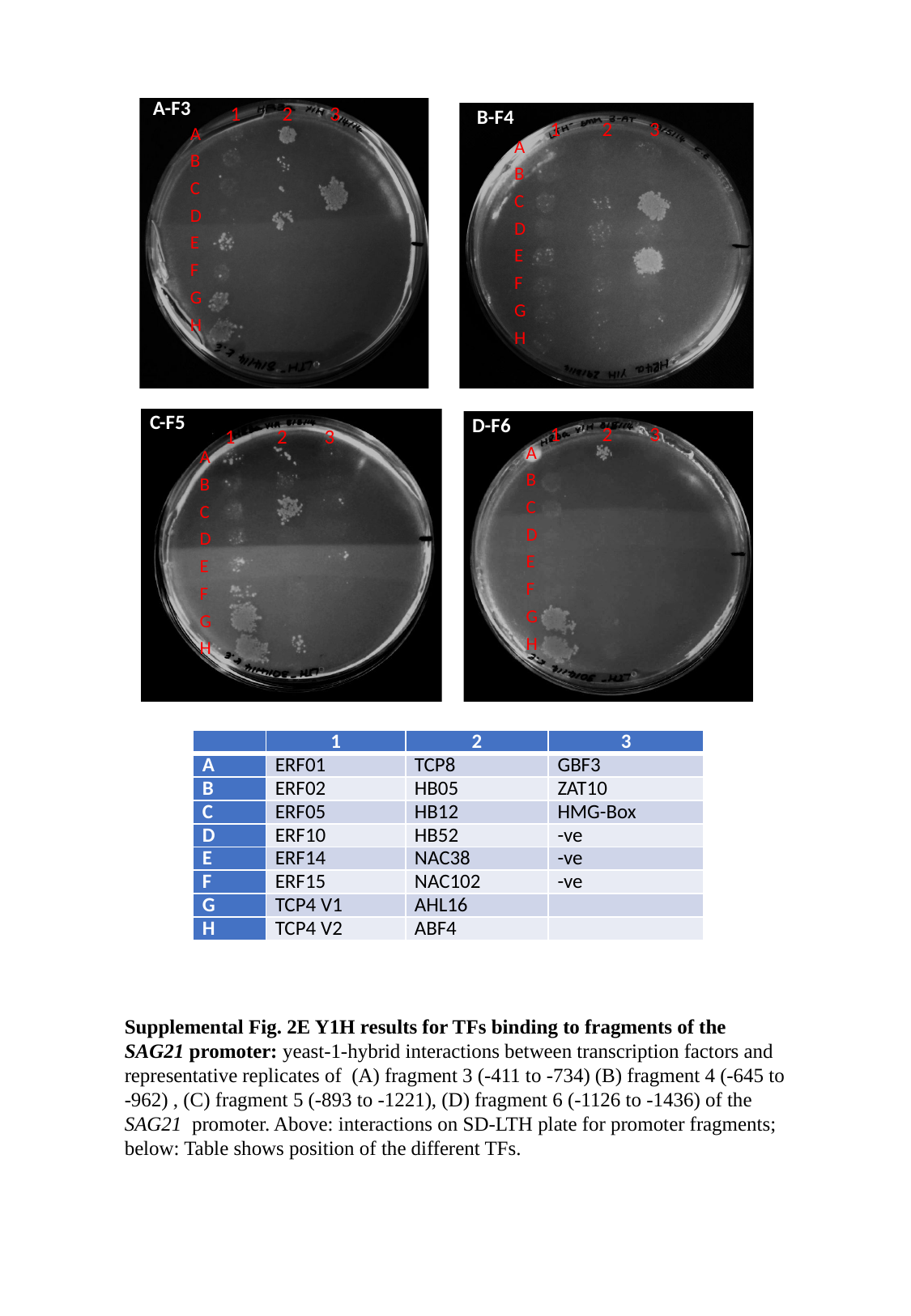

A-F3
1 2 3
B-F4
1 2 3
A
B
C
D
E
F
G
H
A
B
C
D
E
F
G
H
C-F5
D-F6
1 2 3
1 2 3
A
B
C
D
E
F
G
H
A
B
C
D
E
F
G
H
| | 1 | 2 | 3 |
| --- | --- | --- | --- |
| A | ERF01 | TCP8 | GBF3 |
| B | ERF02 | HB05 | ZAT10 |
| C | ERF05 | HB12 | HMG-Box |
| D | ERF10 | HB52 | -ve |
| E | ERF14 | NAC38 | -ve |
| F | ERF15 | NAC102 | -ve |
| G | TCP4 V1 | AHL16 | |
| H | TCP4 V2 | ABF4 | |
Supplemental Fig. 2E Y1H results for TFs binding to fragments of the SAG21 promoter: yeast-1-hybrid interactions between transcription factors and representative replicates of (A) fragment 3 (-411 to -734) (B) fragment 4 (-645 to -962) , (C) fragment 5 (-893 to -1221), (D) fragment 6 (-1126 to -1436) of the SAG21 promoter. Above: interactions on SD-LTH plate for promoter fragments; below: Table shows position of the different TFs.

## Slide 9
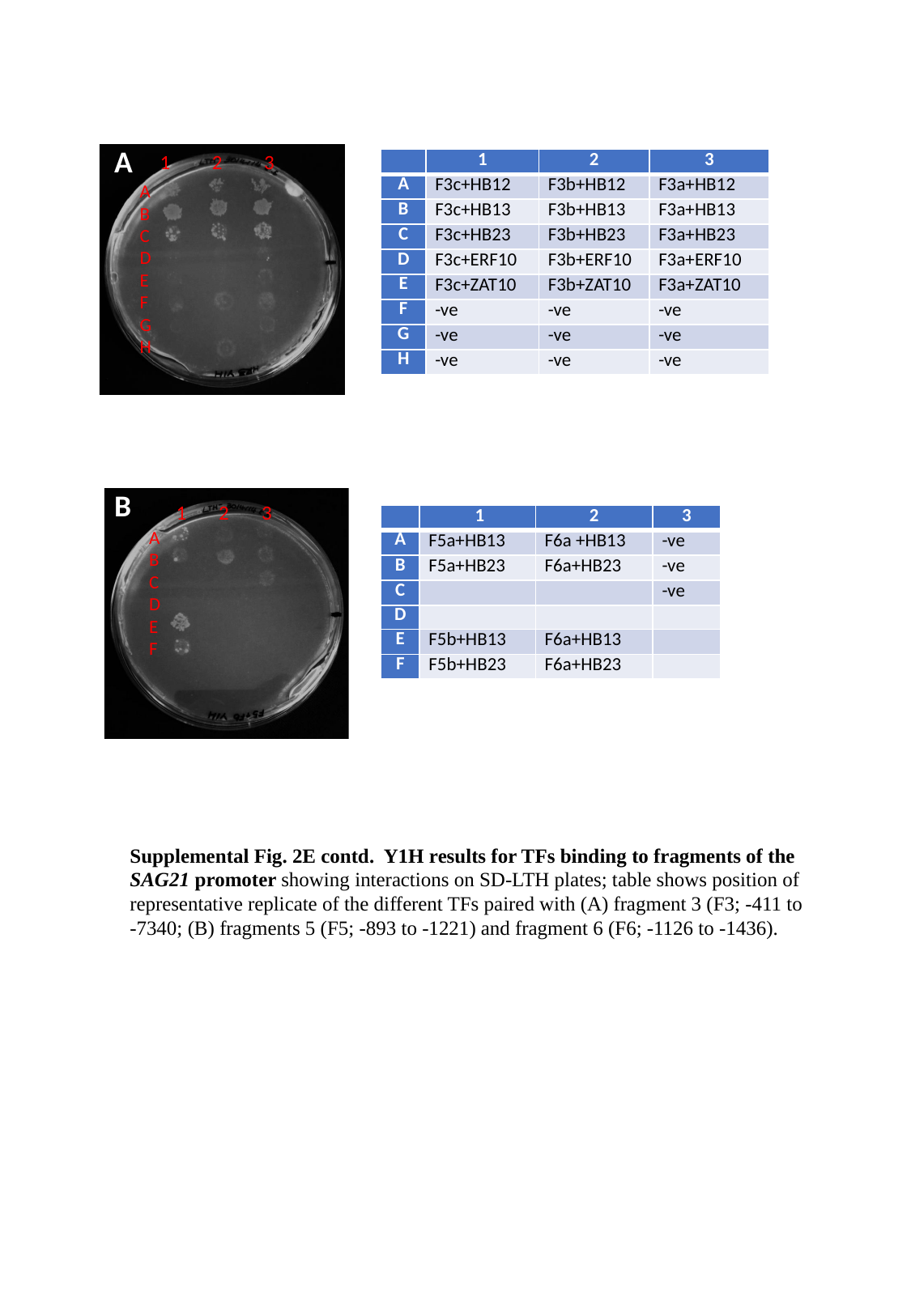

A
1 2 3
| | 1 | 2 | 3 |
| --- | --- | --- | --- |
| A | F3c+HB12 | F3b+HB12 | F3a+HB12 |
| B | F3c+HB13 | F3b+HB13 | F3a+HB13 |
| C | F3c+HB23 | F3b+HB23 | F3a+HB23 |
| D | F3c+ERF10 | F3b+ERF10 | F3a+ERF10 |
| E | F3c+ZAT10 | F3b+ZAT10 | F3a+ZAT10 |
| F | -ve | -ve | -ve |
| G | -ve | -ve | -ve |
| H | -ve | -ve | -ve |
A
B
C
D
E
F
G
H
B
1 2 3
| | 1 | 2 | 3 |
| --- | --- | --- | --- |
| A | F5a+HB13 | F6a +HB13 | -ve |
| B | F5a+HB23 | F6a+HB23 | -ve |
| C | | | -ve |
| D | | | |
| E | F5b+HB13 | F6a+HB13 | |
| F | F5b+HB23 | F6a+HB23 | |
A
B
C
D
E
F
Supplemental Fig. 2E contd. Y1H results for TFs binding to fragments of the SAG21 promoter showing interactions on SD-LTH plates; table shows position of representative replicate of the different TFs paired with (A) fragment 3 (F3; -411 to -7340; (B) fragments 5 (F5; -893 to -1221) and fragment 6 (F6; -1126 to -1436).

## Slide 10
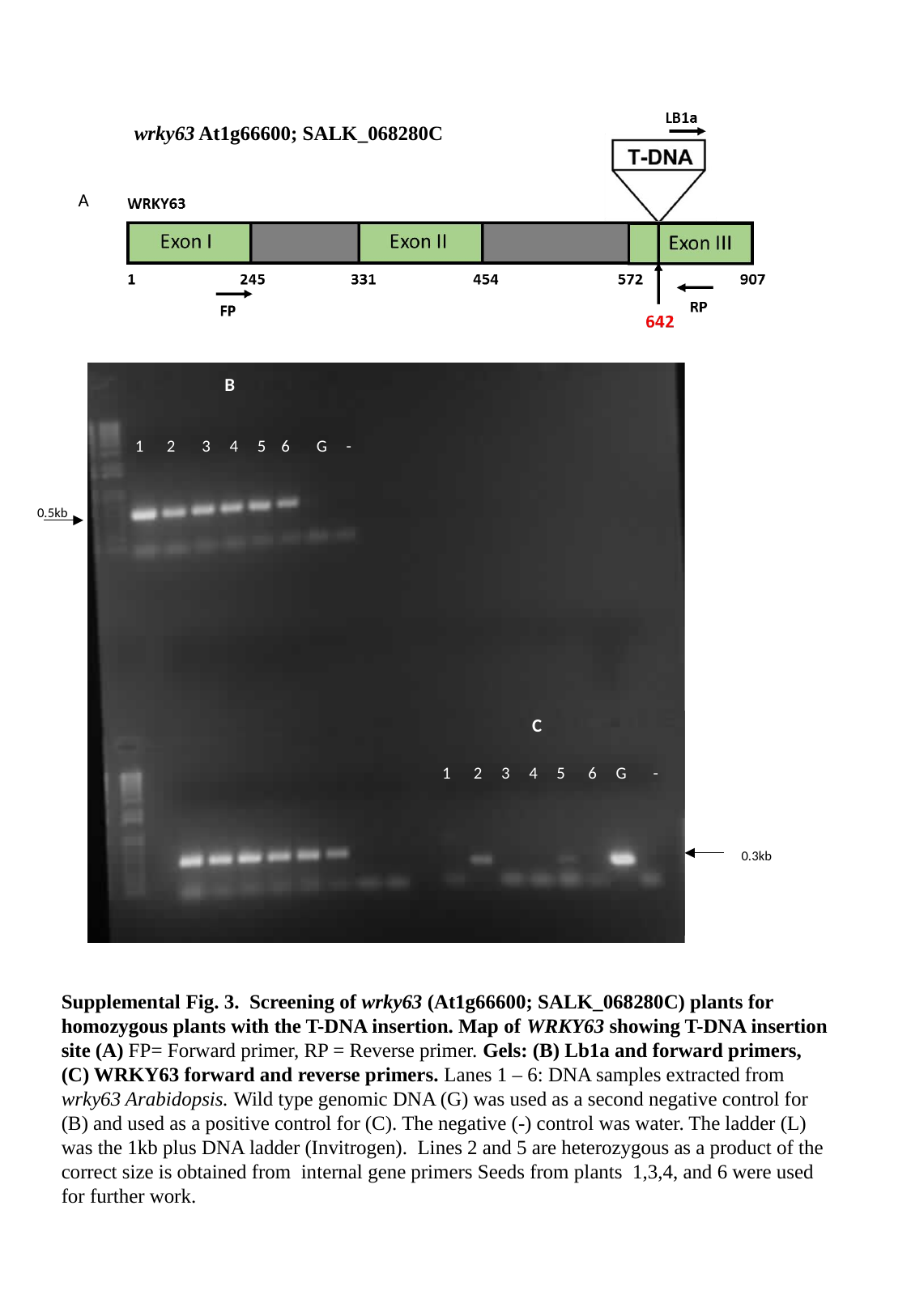

wrky63 At1g66600; SALK_068280C
A
B
 1 2 3 4 5 6 G -
0.5kb
C
C
1 2 3 4 5 6 G -
0.3kb
Supplemental Fig. 3. Screening of wrky63 (At1g66600; SALK_068280C) plants for homozygous plants with the T-DNA insertion. Map of WRKY63 showing T-DNA insertion site (A) FP= Forward primer, RP = Reverse primer. Gels: (B) Lb1a and forward primers, (C) WRKY63 forward and reverse primers. Lanes 1 – 6: DNA samples extracted from wrky63 Arabidopsis. Wild type genomic DNA (G) was used as a second negative control for (B) and used as a positive control for (C). The negative (-) control was water. The ladder (L) was the 1kb plus DNA ladder (Invitrogen). Lines 2 and 5 are heterozygous as a product of the correct size is obtained from internal gene primers Seeds from plants 1,3,4, and 6 were used for further work.

## Slide 11
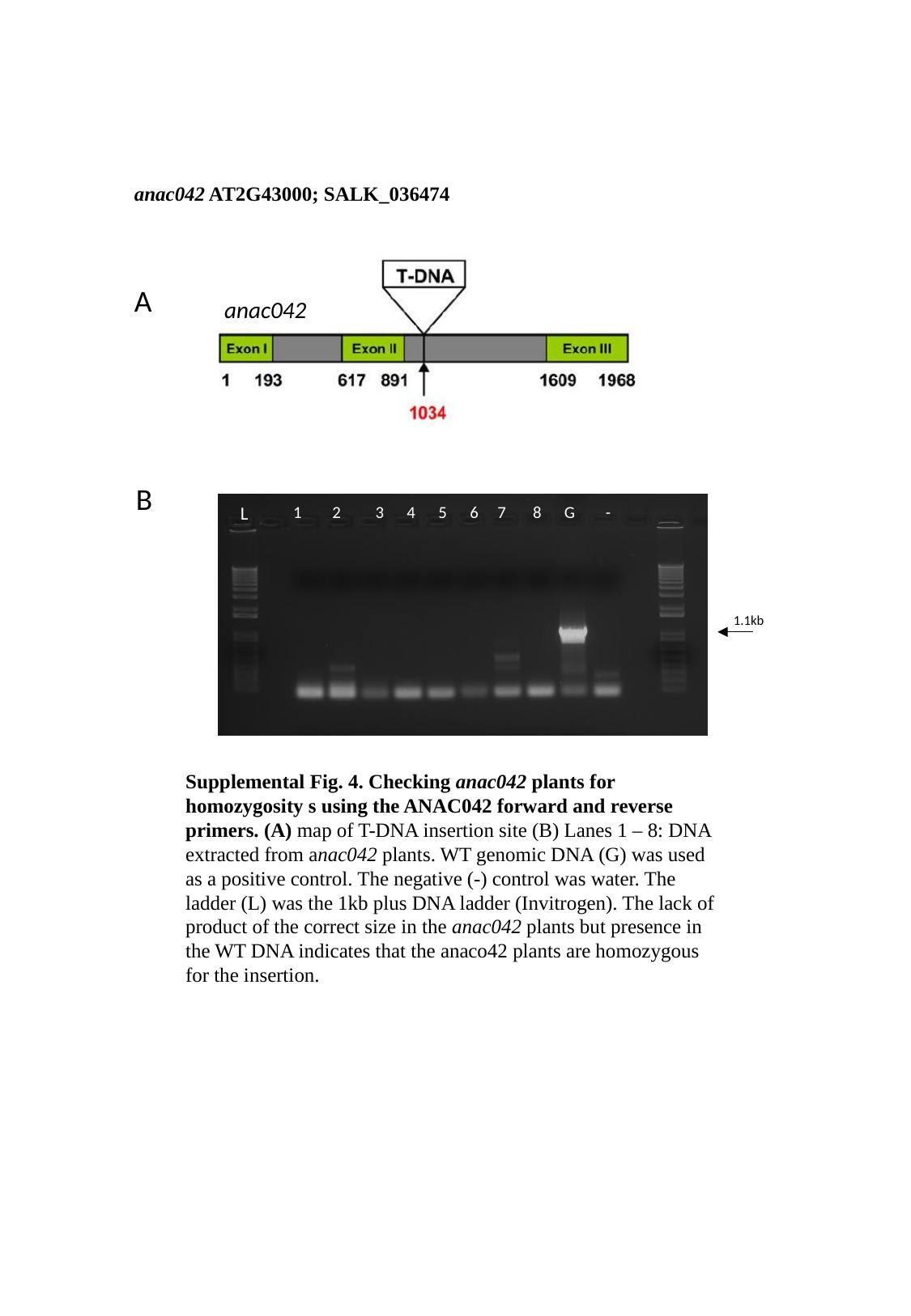

anac042 AT2G43000; SALK_036474
A
anac042
B
L
1 2 3 4 5 6 7 8 G -
1.1kb
Supplemental Fig. 4. Checking anac042 plants for homozygosity s using the ANAC042 forward and reverse primers. (A) map of T-DNA insertion site (B) Lanes 1 – 8: DNA extracted from anac042 plants. WT genomic DNA (G) was used as a positive control. The negative (-) control was water. The ladder (L) was the 1kb plus DNA ladder (Invitrogen). The lack of product of the correct size in the anac042 plants but presence in the WT DNA indicates that the anaco42 plants are homozygous for the insertion.

## Slide 12
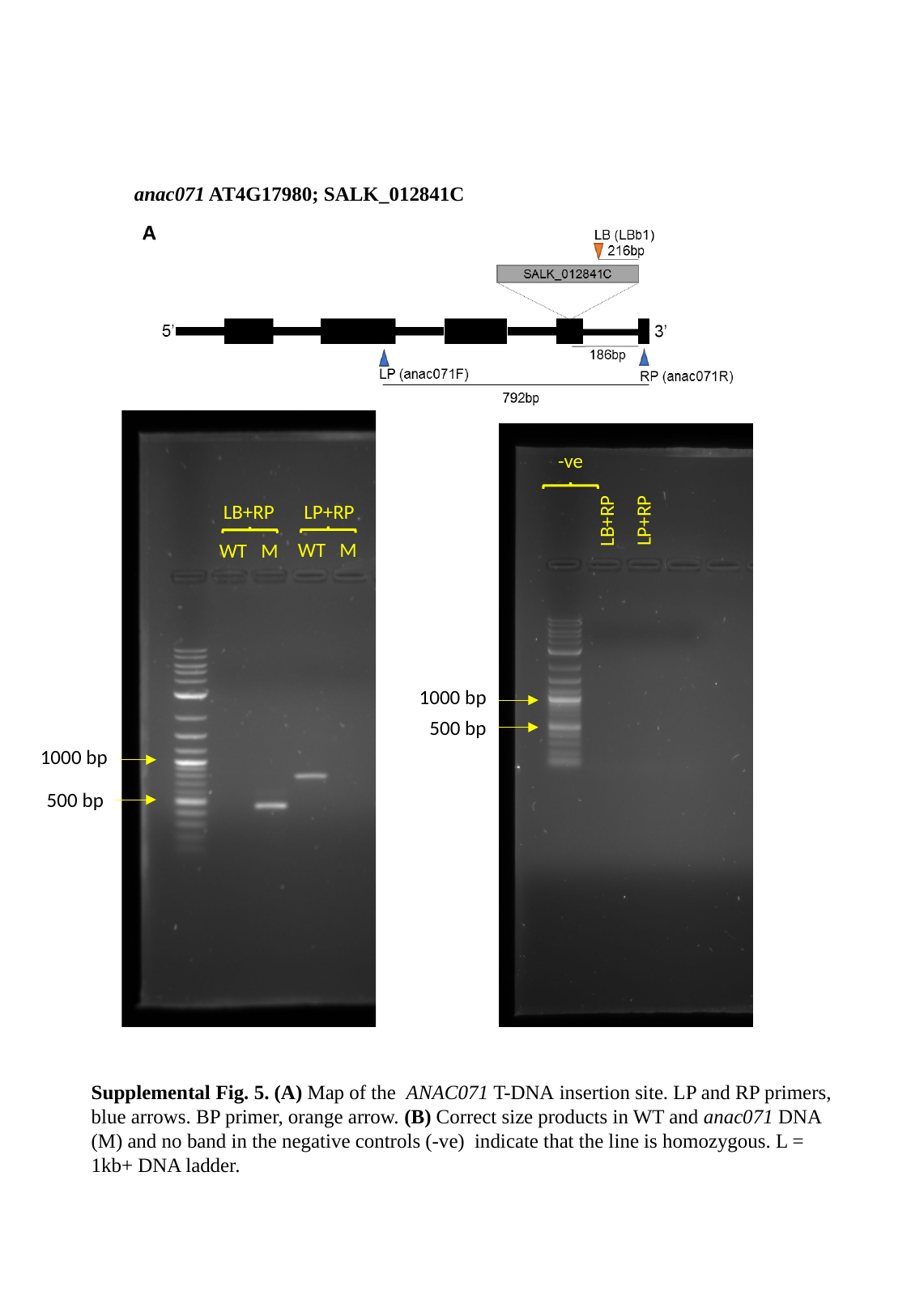

anac071 AT4G17980; SALK_012841C
1000 bp
500 bp
LP+RP
LB+RP
WT M
WT M
LB+RP
LP+RP
1000 bp
500 bp
-ve
Supplemental Fig. 5. (A) Map of the ANAC071 T-DNA insertion site. LP and RP primers, blue arrows. BP primer, orange arrow. (B) Correct size products in WT and anac071 DNA (M) and no band in the negative controls (-ve) indicate that the line is homozygous. L = 1kb+ DNA ladder.

## Slide 13
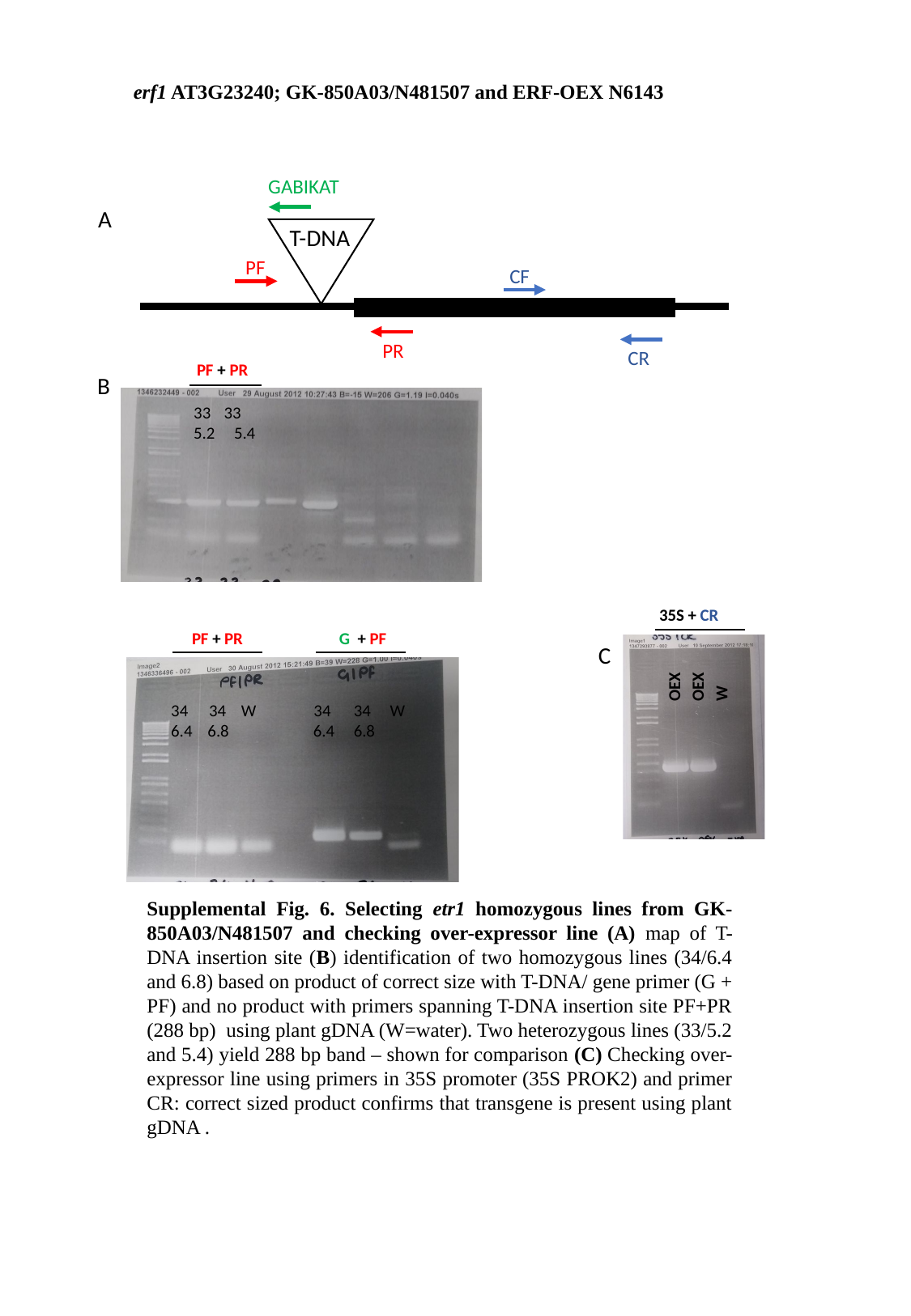

erf1 AT3G23240; GK-850A03/N481507 and ERF-OEX N6143
GABIKAT
A
T-DNA
PF
CF
PR
CR
PF + PR
B
33
5.2 5.4
 35S + CR
PF + PR G + PF
C
OEX
OEX
W
 34 W 34 34 W
6.4 6.8 6.4 6.8
Supplemental Fig. 6. Selecting etr1 homozygous lines from GK-850A03/N481507 and checking over-expressor line (A) map of T-DNA insertion site (B) identification of two homozygous lines (34/6.4 and 6.8) based on product of correct size with T-DNA/ gene primer (G + PF) and no product with primers spanning T-DNA insertion site PF+PR (288 bp) using plant gDNA (W=water). Two heterozygous lines (33/5.2 and 5.4) yield 288 bp band – shown for comparison (C) Checking over-expressor line using primers in 35S promoter (35S PROK2) and primer CR: correct sized product confirms that transgene is present using plant gDNA .

## Slide 14
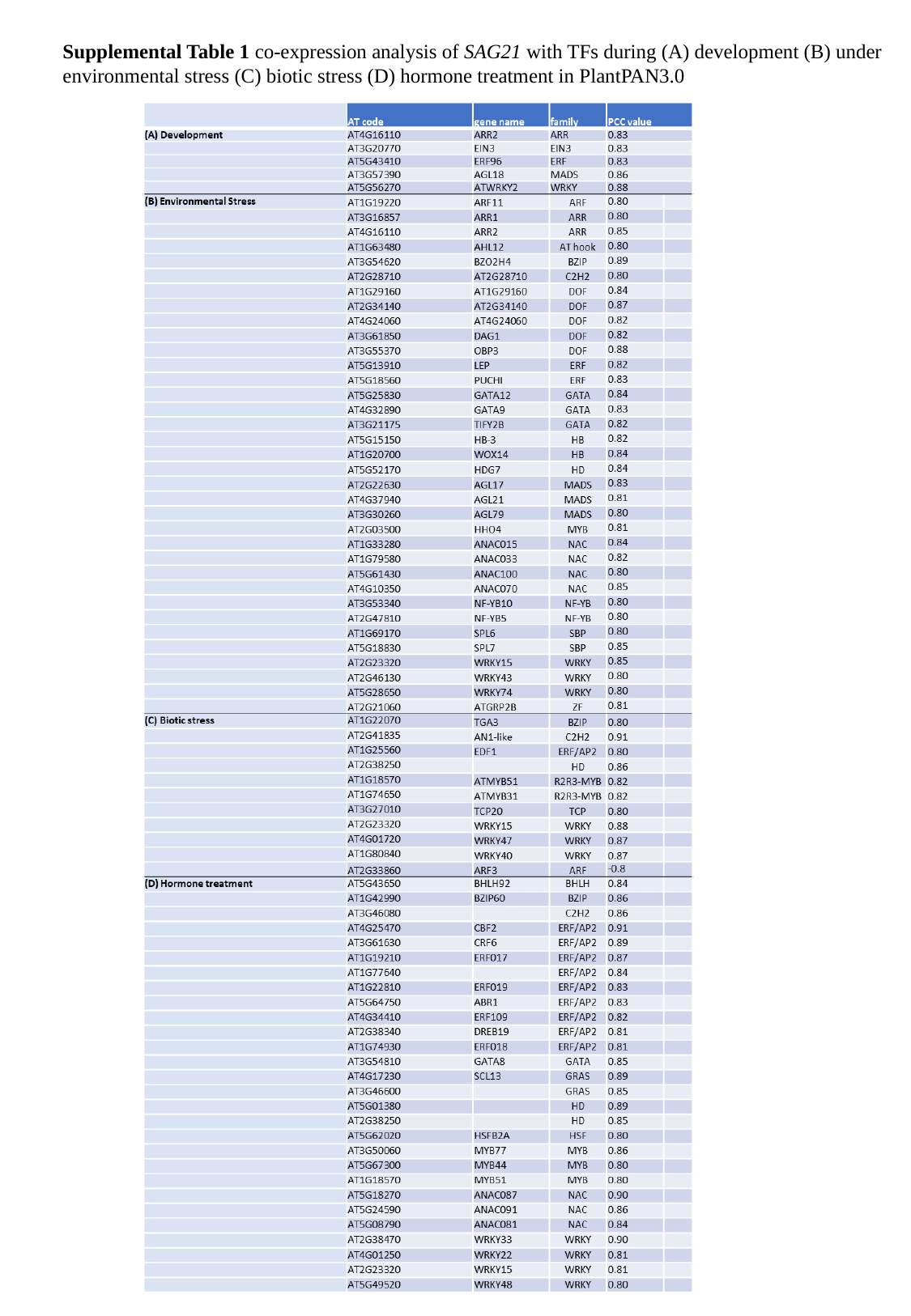

Supplemental Table 1 co-expression analysis of SAG21 with TFs during (A) development (B) under environmental stress (C) biotic stress (D) hormone treatment in PlantPAN3.0
Supplementary Table S1 co-expression analysis of SAG21 with TFs during (A) development (B) under environmental stress (C) biotic stress (D) hormone treatment in PlantPAN3.0

## Slide 15
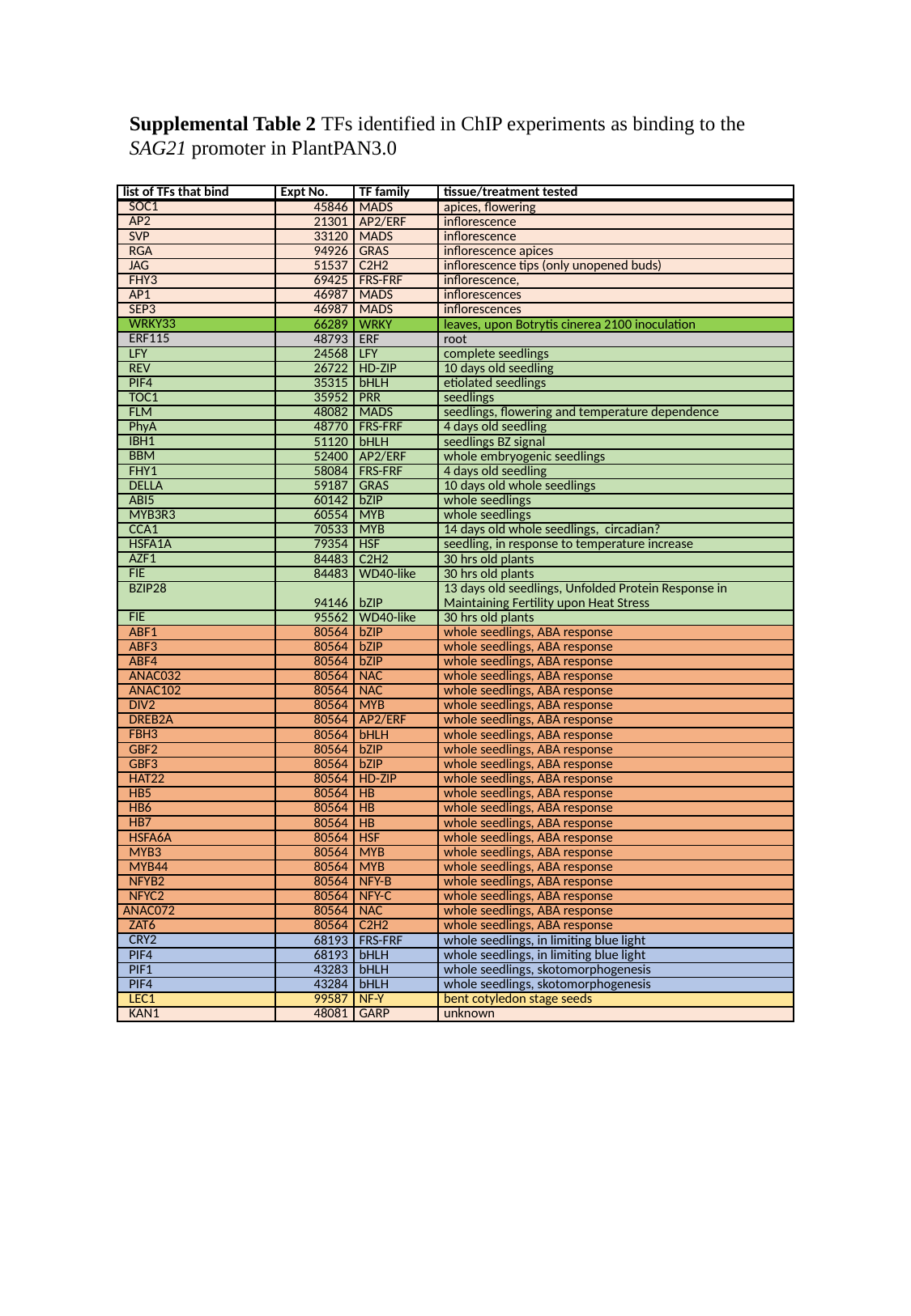

Supplemental Table 2 TFs identified in ChIP experiments as binding to the SAG21 promoter in PlantPAN3.0
| list of TFs that bind | Expt No. | TF family | tissue/treatment tested |
| --- | --- | --- | --- |
| SOC1 | 45846 | MADS | apices, flowering |
| AP2 | 21301 | AP2/ERF | inflorescence |
| SVP | 33120 | MADS | inflorescence |
| RGA | 94926 | GRAS | inflorescence apices |
| JAG | 51537 | C2H2 | inflorescence tips (only unopened buds) |
| FHY3 | 69425 | FRS-FRF | inflorescence, |
| AP1 | 46987 | MADS | inflorescences |
| SEP3 | 46987 | MADS | inflorescences |
| WRKY33 | 66289 | WRKY | leaves, upon Botrytis cinerea 2100 inoculation |
| ERF115 | 48793 | ERF | root |
| LFY | 24568 | LFY | complete seedlings |
| REV | 26722 | HD-ZIP | 10 days old seedling |
| PIF4 | 35315 | bHLH | etiolated seedlings |
| TOC1 | 35952 | PRR | seedlings |
| FLM | 48082 | MADS | seedlings, flowering and temperature dependence |
| PhyA | 48770 | FRS-FRF | 4 days old seedling |
| IBH1 | 51120 | bHLH | seedlings BZ signal |
| BBM | 52400 | AP2/ERF | whole embryogenic seedlings |
| FHY1 | 58084 | FRS-FRF | 4 days old seedling |
| DELLA | 59187 | GRAS | 10 days old whole seedlings |
| ABI5 | 60142 | bZIP | whole seedlings |
| MYB3R3 | 60554 | MYB | whole seedlings |
| CCA1 | 70533 | MYB | 14 days old whole seedlings, circadian? |
| HSFA1A | 79354 | HSF | seedling, in response to temperature increase |
| AZF1 | 84483 | C2H2 | 30 hrs old plants |
| FIE | 84483 | WD40-like | 30 hrs old plants |
| BZIP28 | 94146 | bZIP | 13 days old seedlings, Unfolded Protein Response in Maintaining Fertility upon Heat Stress |
| FIE | 95562 | WD40-like | 30 hrs old plants |
| ABF1 | 80564 | bZIP | whole seedlings, ABA response |
| ABF3 | 80564 | bZIP | whole seedlings, ABA response |
| ABF4 | 80564 | bZIP | whole seedlings, ABA response |
| ANAC032 | 80564 | NAC | whole seedlings, ABA response |
| ANAC102 | 80564 | NAC | whole seedlings, ABA response |
| DIV2 | 80564 | MYB | whole seedlings, ABA response |
| DREB2A | 80564 | AP2/ERF | whole seedlings, ABA response |
| FBH3 | 80564 | bHLH | whole seedlings, ABA response |
| GBF2 | 80564 | bZIP | whole seedlings, ABA response |
| GBF3 | 80564 | bZIP | whole seedlings, ABA response |
| HAT22 | 80564 | HD-ZIP | whole seedlings, ABA response |
| HB5 | 80564 | HB | whole seedlings, ABA response |
| HB6 | 80564 | HB | whole seedlings, ABA response |
| HB7 | 80564 | HB | whole seedlings, ABA response |
| HSFA6A | 80564 | HSF | whole seedlings, ABA response |
| MYB3 | 80564 | MYB | whole seedlings, ABA response |
| MYB44 | 80564 | MYB | whole seedlings, ABA response |
| NFYB2 | 80564 | NFY-B | whole seedlings, ABA response |
| NFYC2 | 80564 | NFY-C | whole seedlings, ABA response |
| ANAC072 | 80564 | NAC | whole seedlings, ABA response |
| ZAT6 | 80564 | C2H2 | whole seedlings, ABA response |
| CRY2 | 68193 | FRS-FRF | whole seedlings, in limiting blue light |
| PIF4 | 68193 | bHLH | whole seedlings, in limiting blue light |
| PIF1 | 43283 | bHLH | whole seedlings, skotomorphogenesis |
| PIF4 | 43284 | bHLH | whole seedlings, skotomorphogenesis |
| LEC1 | 99587 | NF-Y | bent cotyledon stage seeds |
| KAN1 | 48081 | GARP | unknown |

## Slide 16
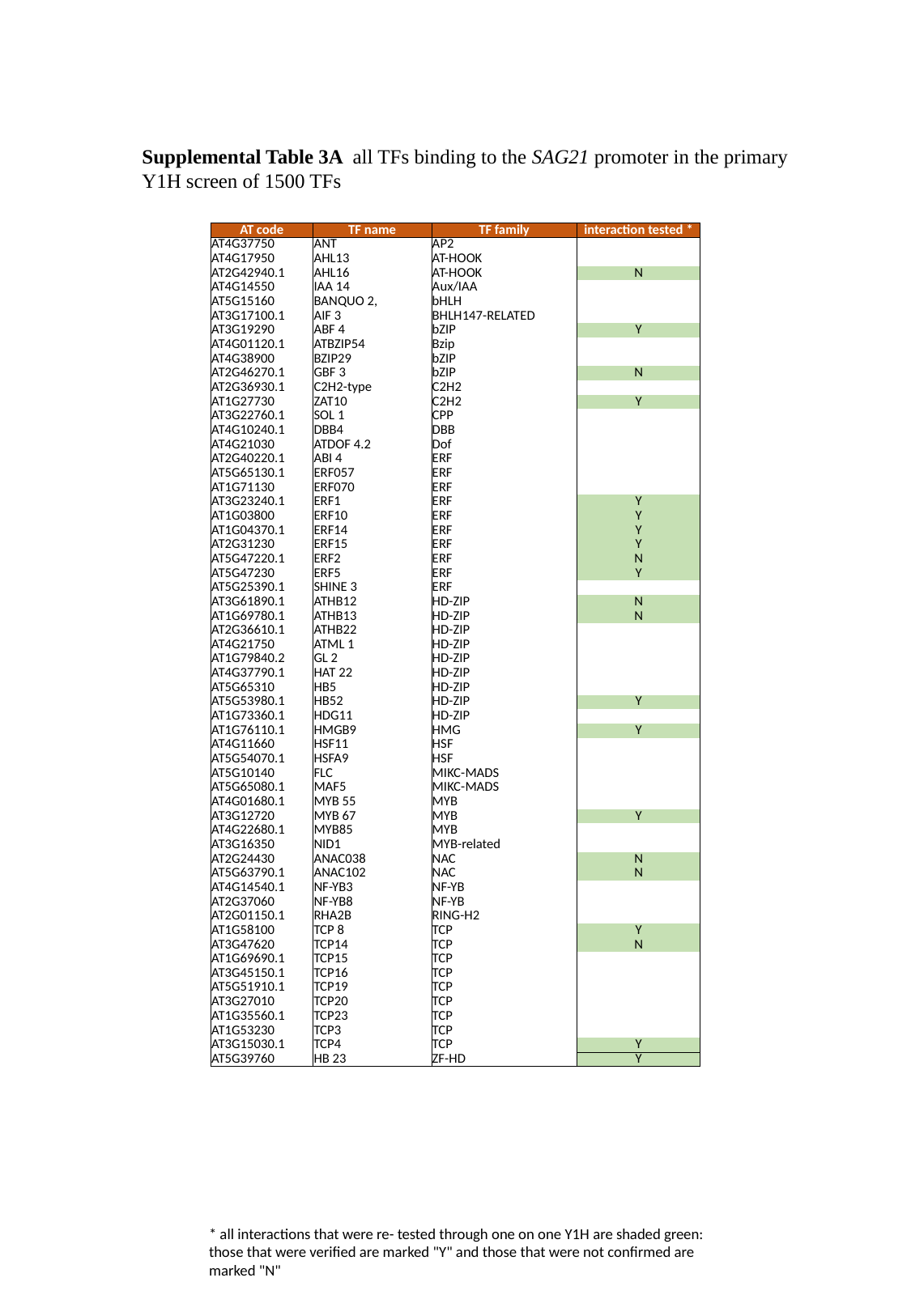

Supplemental Table 3A all TFs binding to the SAG21 promoter in the primary Y1H screen of 1500 TFs
| AT code | TF name | TF family | interaction tested \* |
| --- | --- | --- | --- |
| AT4G37750 | ANT | AP2 | |
| AT4G17950 | AHL13 | AT-HOOK | |
| AT2G42940.1 | AHL16 | AT-HOOK | N |
| AT4G14550 | IAA 14 | Aux/IAA | |
| AT5G15160 | BANQUO 2, | bHLH | |
| AT3G17100.1 | AIF 3 | BHLH147-RELATED | |
| AT3G19290 | ABF 4 | bZIP | Y |
| AT4G01120.1 | ATBZIP54 | Bzip | |
| AT4G38900 | BZIP29 | bZIP | |
| AT2G46270.1 | GBF 3 | bZIP | N |
| AT2G36930.1 | C2H2-type | C2H2 | |
| AT1G27730 | ZAT10 | C2H2 | Y |
| AT3G22760.1 | SOL 1 | CPP | |
| AT4G10240.1 | DBB4 | DBB | |
| AT4G21030 | ATDOF 4.2 | Dof | |
| AT2G40220.1 | ABI 4 | ERF | |
| AT5G65130.1 | ERF057 | ERF | |
| AT1G71130 | ERF070 | ERF | |
| AT3G23240.1 | ERF1 | ERF | Y |
| AT1G03800 | ERF10 | ERF | Y |
| AT1G04370.1 | ERF14 | ERF | Y |
| AT2G31230 | ERF15 | ERF | Y |
| AT5G47220.1 | ERF2 | ERF | N |
| AT5G47230 | ERF5 | ERF | Y |
| AT5G25390.1 | SHINE 3 | ERF | |
| AT3G61890.1 | ATHB12 | HD-ZIP | N |
| AT1G69780.1 | ATHB13 | HD-ZIP | N |
| AT2G36610.1 | ATHB22 | HD-ZIP | |
| AT4G21750 | ATML 1 | HD-ZIP | |
| AT1G79840.2 | GL 2 | HD-ZIP | |
| AT4G37790.1 | HAT 22 | HD-ZIP | |
| AT5G65310 | HB5 | HD-ZIP | |
| AT5G53980.1 | HB52 | HD-ZIP | Y |
| AT1G73360.1 | HDG11 | HD-ZIP | |
| AT1G76110.1 | HMGB9 | HMG | Y |
| AT4G11660 | HSF11 | HSF | |
| AT5G54070.1 | HSFA9 | HSF | |
| AT5G10140 | FLC | MIKC-MADS | |
| AT5G65080.1 | MAF5 | MIKC-MADS | |
| AT4G01680.1 | MYB 55 | MYB | |
| AT3G12720 | MYB 67 | MYB | Y |
| AT4G22680.1 | MYB85 | MYB | |
| AT3G16350 | NID1 | MYB-related | |
| AT2G24430 | ANAC038 | NAC | N |
| AT5G63790.1 | ANAC102 | NAC | N |
| AT4G14540.1 | NF-YB3 | NF-YB | |
| AT2G37060 | NF-YB8 | NF-YB | |
| AT2G01150.1 | RHA2B | RING-H2 | |
| AT1G58100 | TCP 8 | TCP | Y |
| AT3G47620 | TCP14 | TCP | N |
| AT1G69690.1 | TCP15 | TCP | |
| AT3G45150.1 | TCP16 | TCP | |
| AT5G51910.1 | TCP19 | TCP | |
| AT3G27010 | TCP20 | TCP | |
| AT1G35560.1 | TCP23 | TCP | |
| AT1G53230 | TCP3 | TCP | |
| AT3G15030.1 | TCP4 | TCP | Y |
| AT5G39760 | HB 23 | ZF-HD | Y |
* all interactions that were re- tested through one on one Y1H are shaded green: those that were verified are marked "Y" and those that were not confirmed are marked "N"

## Slide 17
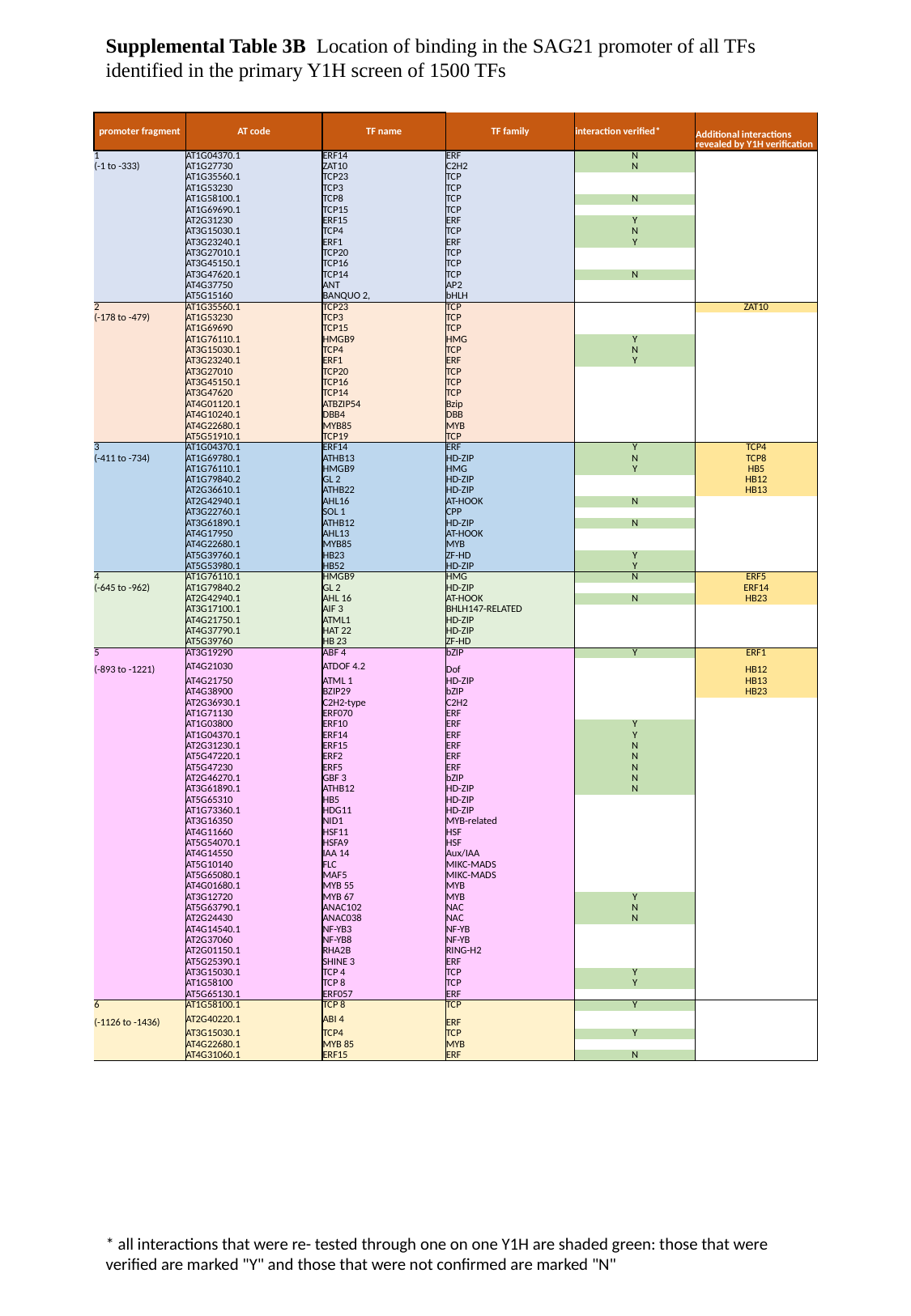

Supplemental Table 3B Location of binding in the SAG21 promoter of all TFs identified in the primary Y1H screen of 1500 TFs
| promoter fragment | AT code | TF name | TF family | interaction verified\* | Additional interactions revealed by Y1H verification |
| --- | --- | --- | --- | --- | --- |
| 1 | AT1G04370.1 | ERF14 | ERF | N | |
| (-1 to -333) | AT1G27730 | ZAT10 | C2H2 | N | |
| | AT1G35560.1 | TCP23 | TCP | | |
| | AT1G53230 | TCP3 | TCP | | |
| | AT1G58100.1 | TCP8 | TCP | N | |
| | AT1G69690.1 | TCP15 | TCP | | |
| | AT2G31230 | ERF15 | ERF | Y | |
| | AT3G15030.1 | TCP4 | TCP | N | |
| | AT3G23240.1 | ERF1 | ERF | Y | |
| | AT3G27010.1 | TCP20 | TCP | | |
| | AT3G45150.1 | TCP16 | TCP | | |
| | AT3G47620.1 | TCP14 | TCP | N | |
| | AT4G37750 | ANT | AP2 | | |
| | AT5G15160 | BANQUO 2, | bHLH | | |
| 2 | AT1G35560.1 | TCP23 | TCP | | ZAT10 |
| (-178 to -479) | AT1G53230 | TCP3 | TCP | | |
| | AT1G69690 | TCP15 | TCP | | |
| | AT1G76110.1 | HMGB9 | HMG | Y | |
| | AT3G15030.1 | TCP4 | TCP | N | |
| | AT3G23240.1 | ERF1 | ERF | Y | |
| | AT3G27010 | TCP20 | TCP | | |
| | AT3G45150.1 | TCP16 | TCP | | |
| | AT3G47620 | TCP14 | TCP | | |
| | AT4G01120.1 | ATBZIP54 | Bzip | | |
| | AT4G10240.1 | DBB4 | DBB | | |
| | AT4G22680.1 | MYB85 | MYB | | |
| | AT5G51910.1 | TCP19 | TCP | | |
| 3 | AT1G04370.1 | ERF14 | ERF | Y | TCP4 |
| (-411 to -734) | AT1G69780.1 | ATHB13 | HD-ZIP | N | TCP8 |
| | AT1G76110.1 | HMGB9 | HMG | Y | HB5 |
| | AT1G79840.2 | GL 2 | HD-ZIP | | HB12 |
| | AT2G36610.1 | ATHB22 | HD-ZIP | | HB13 |
| | AT2G42940.1 | AHL16 | AT-HOOK | N | |
| | AT3G22760.1 | SOL 1 | CPP | | |
| | AT3G61890.1 | ATHB12 | HD-ZIP | N | |
| | AT4G17950 | AHL13 | AT-HOOK | | |
| | AT4G22680.1 | MYB85 | MYB | | |
| | AT5G39760.1 | HB23 | ZF-HD | Y | |
| | AT5G53980.1 | HB52 | HD-ZIP | Y | |
| 4 | AT1G76110.1 | HMGB9 | HMG | N | ERF5 |
| (-645 to -962) | AT1G79840.2 | GL 2 | HD-ZIP | | ERF14 |
| | AT2G42940.1 | AHL 16 | AT-HOOK | N | HB23 |
| | AT3G17100.1 | AIF 3 | BHLH147-RELATED | | |
| | AT4G21750.1 | ATML1 | HD-ZIP | | |
| | AT4G37790.1 | HAT 22 | HD-ZIP | | |
| | AT5G39760 | HB 23 | ZF-HD | | |
| 5 | AT3G19290 | ABF 4 | bZIP | Y | ERF1 |
| (-893 to -1221) | AT4G21030 | ATDOF 4.2 | Dof | | HB12 |
| | AT4G21750 | ATML 1 | HD-ZIP | | HB13 |
| | AT4G38900 | BZIP29 | bZIP | | HB23 |
| | AT2G36930.1 | C2H2-type | C2H2 | | |
| | AT1G71130 | ERF070 | ERF | | |
| | AT1G03800 | ERF10 | ERF | Y | |
| | AT1G04370.1 | ERF14 | ERF | Y | |
| | AT2G31230.1 | ERF15 | ERF | N | |
| | AT5G47220.1 | ERF2 | ERF | N | |
| | AT5G47230 | ERF5 | ERF | N | |
| | AT2G46270.1 | GBF 3 | bZIP | N | |
| | AT3G61890.1 | ATHB12 | HD-ZIP | N | |
| | AT5G65310 | HB5 | HD-ZIP | | |
| | AT1G73360.1 | HDG11 | HD-ZIP | | |
| | AT3G16350 | NID1 | MYB-related | | |
| | AT4G11660 | HSF11 | HSF | | |
| | AT5G54070.1 | HSFA9 | HSF | | |
| | AT4G14550 | IAA 14 | Aux/IAA | | |
| | AT5G10140 | FLC | MIKC-MADS | | |
| | AT5G65080.1 | MAF5 | MIKC-MADS | | |
| | AT4G01680.1 | MYB 55 | MYB | | |
| | AT3G12720 | MYB 67 | MYB | Y | |
| | AT5G63790.1 | ANAC102 | NAC | N | |
| | AT2G24430 | ANAC038 | NAC | N | |
| | AT4G14540.1 | NF-YB3 | NF-YB | | |
| | AT2G37060 | NF-YB8 | NF-YB | | |
| | AT2G01150.1 | RHA2B | RING-H2 | | |
| | AT5G25390.1 | SHINE 3 | ERF | | |
| | AT3G15030.1 | TCP 4 | TCP | Y | |
| | AT1G58100 | TCP 8 | TCP | Y | |
| | AT5G65130.1 | ERF057 | ERF | | |
| 6 | AT1G58100.1 | TCP 8 | TCP | Y | |
| (-1126 to -1436) | AT2G40220.1 | ABI 4 | ERF | | |
| | AT3G15030.1 | TCP4 | TCP | Y | |
| | AT4G22680.1 | MYB 85 | MYB | | |
| | AT4G31060.1 | ERF15 | ERF | N | |
* all interactions that were re- tested through one on one Y1H are shaded green: those that were verified are marked "Y" and those that were not confirmed are marked "N"

## Slide 18
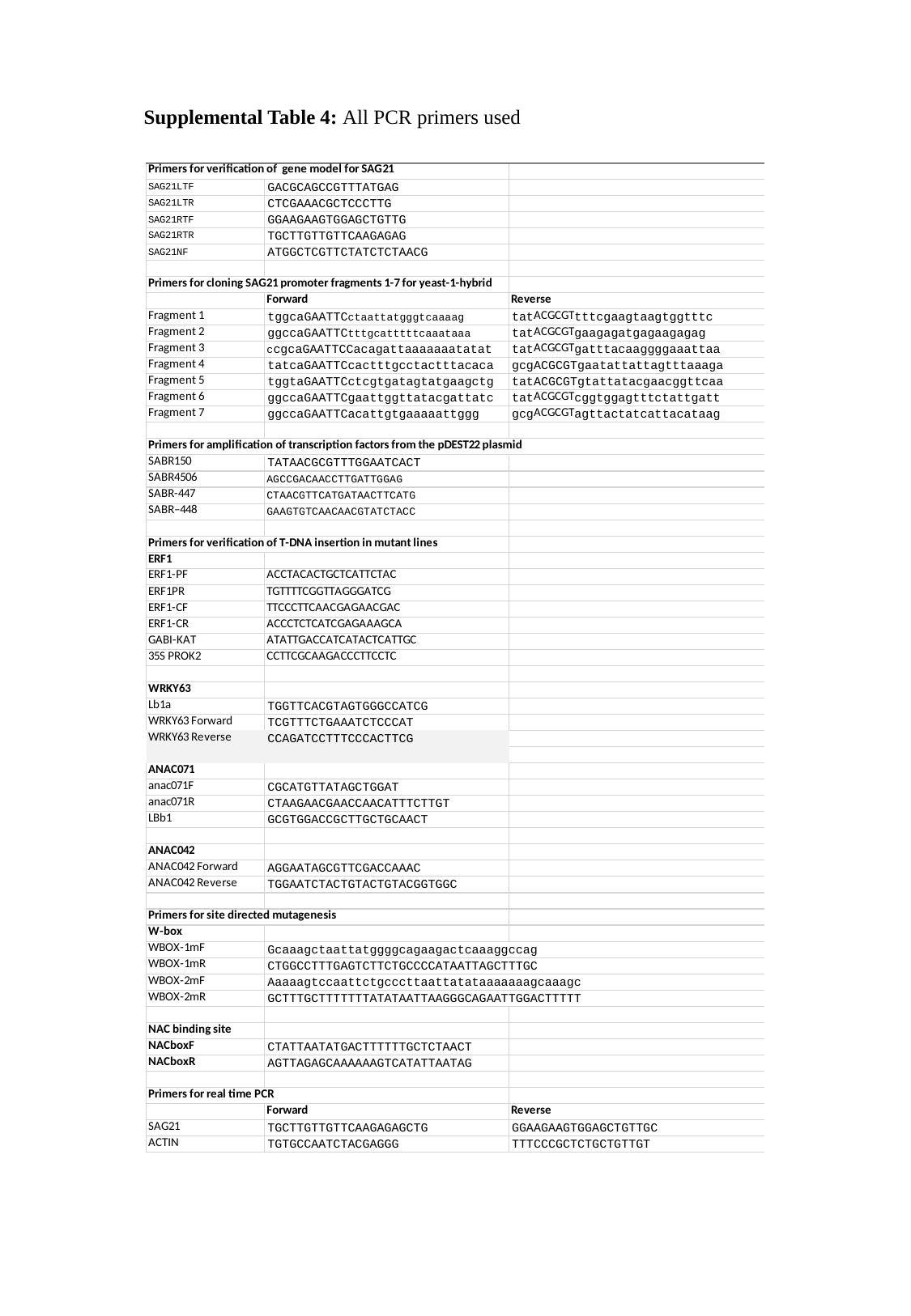

Supplemental Table 4: All PCR primers used

## Slide 19
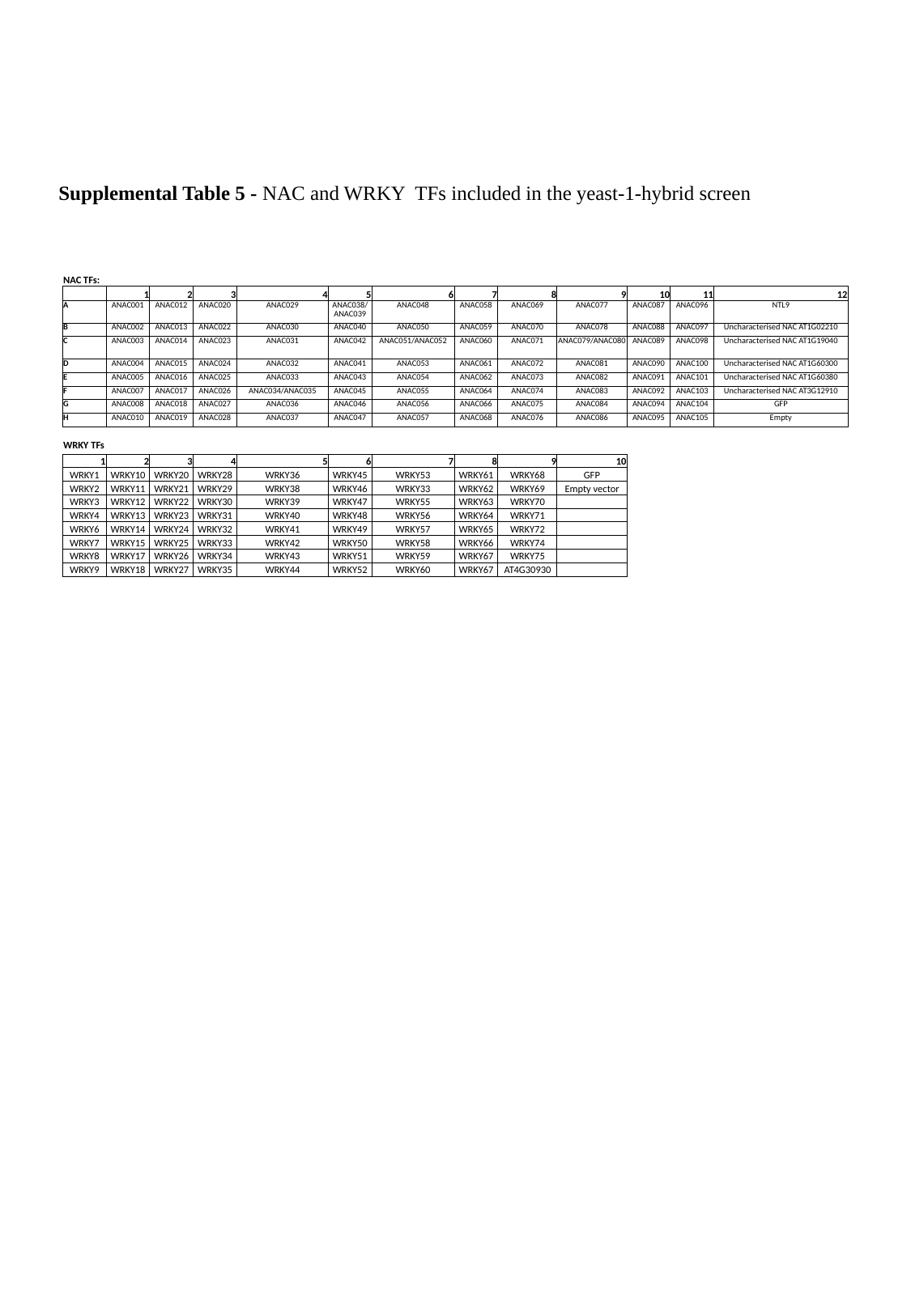

Supplemental Table 5 - NAC and WRKY TFs included in the yeast-1-hybrid screen
| NAC TFs: | | | | | | | | | | | | |
| --- | --- | --- | --- | --- | --- | --- | --- | --- | --- | --- | --- | --- |
| | 1 | 2 | 3 | 4 | 5 | 6 | 7 | 8 | 9 | 10 | 11 | 12 |
| A | ANAC001 | ANAC012 | ANAC020 | ANAC029 | ANAC038/ANAC039 | ANAC048 | ANAC058 | ANAC069 | ANAC077 | ANAC087 | ANAC096 | NTL9 |
| B | ANAC002 | ANAC013 | ANAC022 | ANAC030 | ANAC040 | ANAC050 | ANAC059 | ANAC070 | ANAC078 | ANAC088 | ANAC097 | Uncharacterised NAC AT1G02210 |
| C | ANAC003 | ANAC014 | ANAC023 | ANAC031 | ANAC042 | ANAC051/ANAC052 | ANAC060 | ANAC071 | ANAC079/ANAC080 | ANAC089 | ANAC098 | Uncharacterised NAC AT1G19040 |
| D | ANAC004 | ANAC015 | ANAC024 | ANAC032 | ANAC041 | ANAC053 | ANAC061 | ANAC072 | ANAC081 | ANAC090 | ANAC100 | Uncharacterised NAC AT1G60300 |
| E | ANAC005 | ANAC016 | ANAC025 | ANAC033 | ANAC043 | ANAC054 | ANAC062 | ANAC073 | ANAC082 | ANAC091 | ANAC101 | Uncharacterised NAC AT1G60380 |
| F | ANAC007 | ANAC017 | ANAC026 | ANAC034/ANAC035 | ANAC045 | ANAC055 | ANAC064 | ANAC074 | ANAC083 | ANAC092 | ANAC103 | Uncharacterised NAC AT3G12910 |
| G | ANAC008 | ANAC018 | ANAC027 | ANAC036 | ANAC046 | ANAC056 | ANAC066 | ANAC075 | ANAC084 | ANAC094 | ANAC104 | GFP |
| H | ANAC010 | ANAC019 | ANAC028 | ANAC037 | ANAC047 | ANAC057 | ANAC068 | ANAC076 | ANAC086 | ANAC095 | ANAC105 | Empty |
| | | | | | | | | | | | | |
| WRKY TFs | | | | | | | | | | | | |
| 1 | 2 | 3 | 4 | 5 | 6 | 7 | 8 | 9 | 10 | | | |
| WRKY1 | WRKY10 | WRKY20 | WRKY28 | WRKY36 | WRKY45 | WRKY53 | WRKY61 | WRKY68 | GFP | | | |
| WRKY2 | WRKY11 | WRKY21 | WRKY29 | WRKY38 | WRKY46 | WRKY33 | WRKY62 | WRKY69 | Empty vector | | | |
| WRKY3 | WRKY12 | WRKY22 | WRKY30 | WRKY39 | WRKY47 | WRKY55 | WRKY63 | WRKY70 | | | | |
| WRKY4 | WRKY13 | WRKY23 | WRKY31 | WRKY40 | WRKY48 | WRKY56 | WRKY64 | WRKY71 | | | | |
| WRKY6 | WRKY14 | WRKY24 | WRKY32 | WRKY41 | WRKY49 | WRKY57 | WRKY65 | WRKY72 | | | | |
| WRKY7 | WRKY15 | WRKY25 | WRKY33 | WRKY42 | WRKY50 | WRKY58 | WRKY66 | WRKY74 | | | | |
| WRKY8 | WRKY17 | WRKY26 | WRKY34 | WRKY43 | WRKY51 | WRKY59 | WRKY67 | WRKY75 | | | | |
| WRKY9 | WRKY18 | WRKY27 | WRKY35 | WRKY44 | WRKY52 | WRKY60 | WRKY67 | AT4G30930 | | | | |
